# Abdominopelvic MR to CT registration using a synthetic CT intermediate

**DOI:** 10.1002/acm2.13731

**Published:** 2022-08-03

**Authors:** Jin Uk Heo, Feifei Zhou, Robert Jones, Jiamin Zheng, Xin Song, Pengjiang Qian, Atallah Baydoun, Melanie S. Traughber, Jung‐Wen Kuo, Rose Al Helo, Cheryl Thompson, Norbert Avril, Daniel DeVincent, Harold Hunt, Amit Gupta, Navid Faraji, Michael Z. Kharouta, Arash Kardan, David Bitonte, Christian B. Langmack, Aaron Nelson, Alexandria Kruzer, Min Yao, Jennifer Dorth, John Nakayama, Steven E. Waggoner, Tithi Biswas, Eleanor Harris, Susan Sandstrom, Bryan J. Traughber, Raymond F. Muzic

**Affiliations:** ^1^ Department of Radiology Case Western Reserve University Cleveland Ohio USA; ^2^ Department of Biomedical Engineering Case Western Reserve University Cleveland Ohio USA; ^3^ Department of Radiology University Hospitals Cleveland Medical Center Cleveland Ohio USA; ^4^ School of Artificial Intelligence and Computer Science Jiangnan University Wuxi Jiangsu China; ^5^ Department of Internal Medicine Louis Stokes Cleveland VA Medical Center Cleveland Ohio USA; ^6^ Department of Radiation Oncology Penn State University Hershey Pennsylvania USA; ^7^ Department of Public Health Sciences Penn State College of Medicine Hershey Pennsylvania USA; ^8^ Department of Radiation Oncology University Hospitals Cleveland Medical Center Cleveland Ohio USA; ^9^ MIM Software Inc. Cleveland Ohio USA; ^10^ Department of Radiation Oncology Case Western Reserve University Cleveland Ohio USA; ^11^ Department of Obstetrics and Gynecology Allegheny Health Network Pittsburgh Pennsylvania USA; ^12^ Department of Obstetrics and Gynecology Cleveland Clinic Cleveland Ohio USA

**Keywords:** local phase difference, multimodality deformable image registration, mutual information, synthetic CT

## Abstract

Accurate coregistration of computed tomography (CT) and magnetic resonance (MR) imaging can provide clinically relevant and complementary information and can serve to facilitate multiple clinical tasks including surgical and radiation treatment planning, and generating a virtual Positron Emission Tomography (PET)/MR for the sites that do not have a PET/MR system available. Despite the long‐standing interest in multimodality co‐registration, a robust, routine clinical solution remains an unmet need. Part of the challenge may be the use of mutual information (MI) maximization and local phase difference (LPD) as similarity metrics, which have limited robustness, efficiency, and are difficult to optimize. Accordingly, we propose registering MR to CT by mapping the MR to a synthetic CT intermediate (sCT) and further using it in a sCT‐CT deformable image registration (DIR) that minimizes the sum of squared differences. The resultant deformation field of a sCT‐CT DIR is applied to the MRI to register it with the CT. Twenty‐five sets of abdominopelvic imaging data are used for evaluation. The proposed method is compared to standard MI‐ and LPD‐based methods, and the multimodality DIR provided by a state of the art, commercially available FDA‐cleared clinical software package. The results are compared using global similarity metrics, Modified Hausdorff Distance, and Dice Similarity Index on six structures. Further, four physicians visually assessed and scored registered images for their registration accuracy. As evident from both quantitative and qualitative evaluation, the proposed method achieved registration accuracy superior to LPD‐ and MI‐based methods and can refine the results of the commercial package DIR when using its results as a starting point. Supported by these, this manuscript concludes the proposed registration method is more robust, accurate, and efficient than the MI‐ and LPD‐based methods.

## 1 INTRODUCTION

Multimodality fusion, the superposition of different image volumes, makes it possible to simultaneously visualize features from different imaging modalities and provide clinically relevant and complementary information, provided that the volumes are accurately coregistered.^1^ In clinical applications, both magnetic resonance (MR) and computed tomography (CT) typically provide anatomic information that can be combined to improve the accuracy of diagnoses and precision of image‐guided surgical and radiation treatment planning. In the latter application, MR leverages superior soft tissue contrast that CT lacks, while CT provides the necessary electron density map needed to calculate the radiation dose.^2^ To be clinically useful for such application, MR and CT spatial agreement should be within 1–2 mm.^3^ As there is no clinically available hybrid MR/CT at present, data must be acquired on separate scanners. Thus, the patient position inevitably differs between modalities, and spatial registration of the image volumes is a prerequisite prior to fusion. Another application where MR‐CT registration is valuable is in creating a “virtual PET/MR.” Given the technical challenges and expense of Positron Emission Tomography (PET)/MR scanners,^4^ the value of combined PET/MR images^5^, ^6^, ^7^, ^8^, ^9^, ^10^ motivates the development of a virtual PET/MR function by registration of MR data to the CT component of PET/CT.

Due to the differences in voxel information, MR to CT registration cannot directly utilize metrics such as the sum of squared differences (SSDs) or cross‐correlation (CC) to optimize the spatial correspondences.^11^ As such, methods using metrics like mutual information (MI), local phase difference (LPD), and their variants, have been investigated.^12^, ^13^, ^14^, ^15^, ^16^ MI‐based registration maximizes shared intensity information between the two images.^12^, ^14^, ^17^ Although MI is widely used in multimodal registration, it has some shortcomings including: (1) slow convergence rate due to discrete joint histogram and the lack of use of gradient‐based optimization methods,^18^ (2) difficulty in finding the global optimum, (3) low accuracy due to lack of spatial information in the intrinsically global measure of MI,^1^, ^19^, ^20^ and (4) misregistration due to small overlapping regions.^1^, ^21^, ^22^ Much effort has been devoted in meeting the above challenges by introducing a differentiable MI to use the gradient‐based optimization,^18^ by including the spatial information for achieving a higher accuracy than the conventional global MI,^1^, ^19^, ^20^ and by proposing a normalized MI to address the partial overlapping issue.^21^ Despite these efforts, the multimodality registration based on MI is still not as robust and efficient as the same‐modality registration, especially for the group‐wise image registration, and thus has limited applicability for routine clinical use.^22^, ^23^

Minimizing local phase difference (LPD) as a similarity metric for multimodality registration is relatively newer as compared to MI approach. Janssens^16^ implemented the LPD method by minimizing the sum of multifrequency and voxel‐wise phase differences between images. Although fast and insensitive to wide intensity variations, misregistration may still occur due to false minima. Other phase‐based methods include the use of phase MI,^24^, ^25^ local phase‐coherence,^26^ and complex phase order.^27^, ^28^ While the registration accuracy could be improved compared with LPD, the implementation of these registration methods still suffers from sophisticated similarity calculation and inefficient optimization.^29^

Recently, some methods for registering MR to CT have been described by transforming the multimodality registration to a same‐modality matching problem in order to achieve more robust multimodality image registration.^1^, ^22^, ^29^, ^30^, ^31^, ^32^, ^33^ However, the actual application and performance evaluation has been scarcely reported. In one study, Roy et al.^30^ generated the sCT using the atlas‐based method with 10 sets of brain MR and CT data, then registered the sCT to the measured CT (mCT) using CC as the similarity metric. In a more recent study, Mckenzie et al.^33^ generated the sCT using CycleGAN and 25 head‐and‐neck volumes, and registered the sCT to the mCT using MI as the similarity metric. The other reports of which we are aware that use sCT also use synthetic MR (sMR); they work by simultaneously registering the sMR to the measured MR and the sCT to the mCT. In this regard, they require additional computation compared to what we are proposing.^29^, ^30^, ^31^, ^32^, ^33^ Surprisingly, all but one of these^29^ used either MI or CC as the similarity metric, which have suboptimal performance and are more challenging to optimize than the SSD.

Herein, we propose a method of using sCT to facilitate MR to CT registration in the abdominopelvic region, which is a more challenging anatomy than the brain. Furthermore, the SSD similarity metric used is computationally more efficient than CC and MI and more robust for automatic optimization than CC and MI, which often have a global maximum within a relatively narrow, peaked region, and the area beyond the neighborhood is relatively flat making it challenging to find.^34^, ^35^, ^36^ In brief, an sCT is created by converting the modified Dixon (mDixon)‐reconstructed MR image volume to CT‐like contrast using a voxel‐wise intensity and segmentation‐based method.^37^ Next, the sCT, derived from MR and thus having exact spatial correspondence to it, is deformably registered to the mCT. Finally, the resultant deformation field is applied to the MR so that it becomes coregistered with the mCT. In the following, Section 2 presents our registration method and performance evaluation protocol. Section 3 presents both quantitative and qualitative results to illustrate the performance of using sCT as an intermediate in MR‐CT registration, and the efficacy of our method in clinical applications. Section 4 presents discussion, and Section 5 is the conclusion.

## 2 MATERIAL AND METHODS

### 2.1 Overview

**FIGURE 1:**
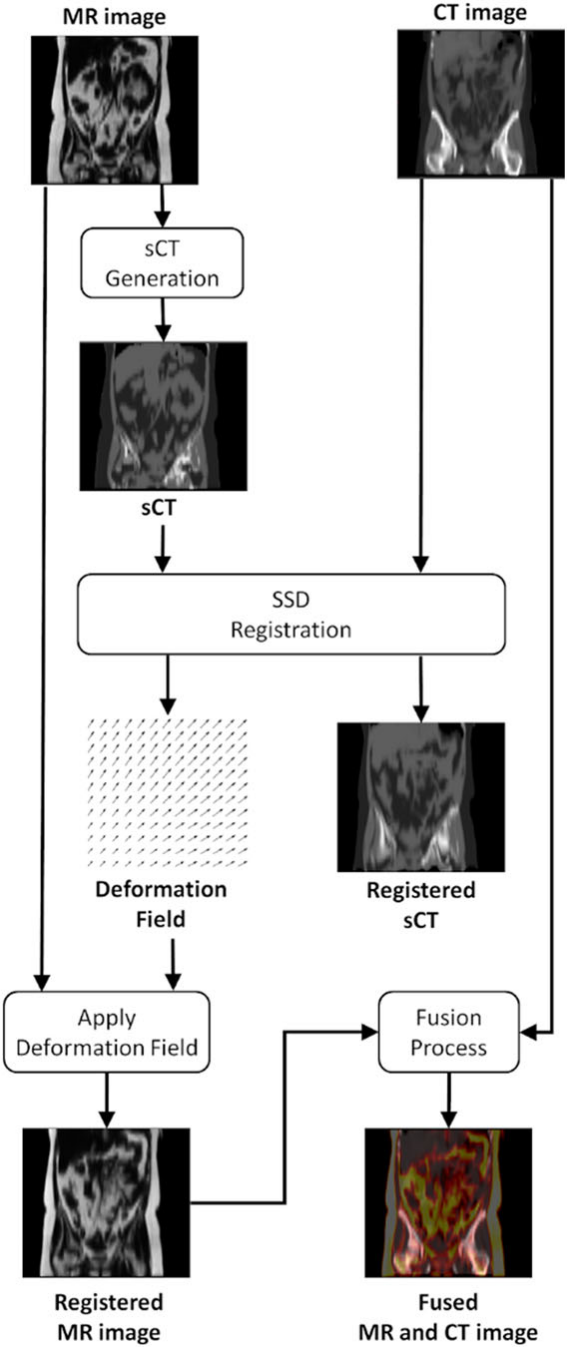
Framework of our proposed method in registering magnetic resonance (MR) with computed tomography (CT).

Figure 1 shows the framework of our method. In brief, machine learning is used to map an MR image volume to an sCT image volume. Then deformable image registration (DIR) is used to bring the sCT into spatial correspondence with the mCT by minimizing the SSD. The resultant deformation field is then used to spatially map the MR to coregister it with the CT. This method is compared to conventional MI and LPD approaches and the DIR provided by the MIM Maestro (version 7.0.66.6.10, MIM Software Inc., Cleveland, OH) software for direct MR to CT registration.^15^, ^16^ MIM Maestro is chosen for comparison since it is widely used, state‐of‐the‐art clinical tool that has Food and Drug Administration (FDA)‐clearance. Registration performance is evaluated using both quantitative metrics and visual assessment of the registration by physicians.

### 2.2 Data acquisition

Volunteers are recruited from oncologic patients undergoing routine clinical PET/CT scanning using a Philips GEMINI TF PET/CT or its Big Bore variant.^38^ This procedure is immediately followed by PET/MR scanning using Philips Ingenuity TF PET/MR.^39^, ^40^ The low dose CT from the PET/CT and the 3T MR images from the PET/MR are used for the present study. Neither scan entails contrast injection. The CT acquisitions use a low‐dose, 120‐kV protocol and are reconstructed to a voxel spacing of 1.17 × 1.17 × 5.00 mm^3^. The MR acquisitions utilize a spin echo imaging pulse sequence to support mDixon reconstruction^41^: Repetition Time (TR) = 3.08 ms, Echo Time (TE) = 1.035 and 1.887 ms. Fat, water, in‐phase (IP), and opposed‐phase (OP) images with a voxel spacing 0.98 × 0.98 × 5.00 mm^3^ are reconstructed. Both CT and MR volumes use 512 × 512 × n matrices, where n depends on the subject size. Twenty‐five sets of whole‐body MR and CT data are acquired.

The subject recruitment, data collection, and management are in compliance with protocols reviewed and approved by the University Hospitals Cleveland Medical Center Institutional Review Board.

### 2.3 Generation of synthetic CT

Synthetic CT images are generated using the Transfer Fuzzy Clustering and Active Learning‐based Classification algorithm (TFC‐ALC) as our group has demonstrated previously.^37^, ^41^, ^42^, ^43^, ^44^, ^45^ In brief, from the cropped MR data, the local texture features are extracted for each of MR water, fat, IP, and OP images by convolving with a 3 × 3 weighting matrix. Combining these four local texture features and the three‐dimensional (3D) position of voxel using grid partition strategy,^37^ a total of seven features are used to classify tissue types for each voxel. This seven‐dimensional feature vector for all the voxels is called a feature map. From the feature map, referenced class prototypes for four different tissue types—bone, air, fat, and soft tissue—can be determined. Candidate voxels for a bone cluster are defined based on CT values exceeding 300 HU. The remaining voxels are analyzed using conventional Fuzzy C‐Means (FCMs) to determine cluster centroids corresponding to three tissue types: air, fat, and soft tissue. The bone cluster centroid is calculated independently as the mean values of the components of the seven‐dimensional features of the candidate bone voxels. Due to the fact that we are leveraging the information from CT to construct FCM models, this particular FCM model is referred to as knowledge‐leveraged transfer fuzzy c‐means (KL‐TFCM). However, at this point, due to a lack of adequate information from the MR images, the cluster centroids of air and bone are not separated sufficiently to reliably distinguish air and bone so resulting in cross‐contamination that will be subsequently addressed. Regardless, this process of obtaining the four cluster centroids is repeated for each subject, and then the referenced class prototypes of bone, fat, air, and soft tissue are generated by averaging all subjects’ centroids.

To resolve the contamination of the bone class by air voxels and the air class by bone voxels, additional analysis is applied during training.^37^, ^44^, ^45^ For each subject, the feature map is partitioned into four clusters with referenced class prototypes obtained from the previous procedure. The fat voxels are satisfactorily distinguished given that mDixon sequences can robustly identify the fat tissue. Then, our active learning‐based support vector machine (AL‐SVM) method^37^ is trained to separate the remaining three classes (bone, air and soft tissue). The predicted labels of AL‐SVM are corrected using the paired CT image. The group of bone and air are refined by separating the bone voxels from the voxels that are wrongly partitioned to air using CT. The rest of the voxels are assigned as soft tissue. This training procedure is repeated for all subjects to obtain the same number of AL‐SVM models with the number of subjects. Combining the fat classifier using KL‐TFCM and the remaining three classifiers using AL‐SVM, the tissue‐distinguishable‐operators (TDOs) are obtained for classifying four tissue types.

For sCT generation, the tissue types of voxels in the target abdominopelvic MR images are distinguished using the voting strategy based on the predicted results from the obtained TDOs and the k‐nearest neighbors algorithm. With the final predicted given voxel labels, the appropriate CT values for each corresponding tissue type are assigned, and a Gaussian filter, with 2.5 mm full‐width‐at‐half‐maximum, is applied to reconstruct a synthetic CT image for the target MR images. Full details are given in our previous publication.^37^

### 2.4 MR to CT registration

The MR and CT data are preprocessed as follows: A rigid registration is performed by aligning the CT image to the MR image using MIM Maestro. Its rigid multimodality image registration utilizes MI, but deformable multimodality image registration utilizes proprietary metrics that are not the conventional similarity metrics such as SSD, CC, or MI (personal communication with MIM Software, Inc). These rigid registered data sets henceforth are referred to as the baseline images and are used as an initial guess (but still is an inadequate registration as will become evident in results). The MR data have a matrix size of 512 × 512 × m with voxel spacing of 0.98 × 0.98 × 5.00 mm^3^. To cover the intended abdominopelvic region, the data were cropped so that the number of slices, m, ranged between 76 and 104, spanning from approximately 2 cm above the apex of the liver to approximately 1 cm below the symphysis pubis. The CT data are then resampled and cropped to the same dimensions. Patients’ arms are removed from both MR and CT images because MR and CT images are scanned from two separate scanners at different times making it extremely difficult to obtain spatially concordant MR‐CT pairs for arms. Further, while arms above the head is the clinically preferred posture, arms along the sides are allowable for people who find the position to be uncomfortable, especially in the PET/MR, which has a limited bore diameter. Such differences may cause difficulty in not only training but also validation of the quality of sCT and registration accuracy so they are removed, noting that the arms are generally not considered as being part of the abdominopelvic region for diagnostic purposes. Likewise, the scanner table is removed from the CT images, whereas no additional processing is needed for the MR data since the table does not generate MR signal.

Of the available MR data, the Dixon OP sequence image is used for all registration and evaluation procedures as it provides enhanced contrast in the form of a distinct black line around organs due to the phase‐cancellation effect between fat and water,^46^, ^47^ and, from the available image sequences (Dixon water, fat, and IP sequences), achieves the most accurate contours for assessing the landmark errors.

For this study, a total of six different image registration methods are evaluated: (1) Rigid registration using MIM Maestro (Baseline image), (2) LPD minimization, (3) MI maximization, (4) SSD minimization (our proposed method), (5) multimodality DIR provided by MIM Maestro default method, and (6) applying method E followed by D (MIM Maestro + SSD‐based method). Table 1 summarizes method name, label, deformation method with optimization metric. For all the methods, the mCT image is used as the fixed image, and the MR image or sCT is used as the moving image.

**TABLE 1:** Summary of all comparing image registration methods with method labels used throughout the presented work

Method name	Rigid registration method/baseline images	LPD‐based method	MI‐based method	SSD‐based method	MIM Maestro default method	MIM Maestro + SSD‐based method
Method label	A	B	C	D	E	F
Deformable	N	Y	Y	Y	Y	Y
Optimization metric	Maestro proprietary	Local phase difference (LPD)	Mutual information (MI)	Sum of squared difference (SSD)	MIM Maestro proprietary	MIM Maestro proprietary + SSD
Interpolation method for image warping	Linear interpolation	Linear interpolation	Linear interpolation	Linear interpolation	Linear interpolation	Linear interpolation
Field computation	Translation + rotation	Morphons	Block Matching	Block matching	MIM Maestro proprietary	MIM Maestro proprietary + Block Matching

In detail, **method A** uses MIM Maestro to perform rigid registration with translation and rotation based on optimizing MI. This is taken as the starting point for all other methods. **Method B** uses OpenREGGUI to perform DIR with morphons^11^, ^48^ algorithm as field computation with minimizing the LPD as the optimization metric. **Method C** uses OpenREGGUI to perform DIR with block matching algorithm^49^ as field computation with maximizing the MI as the optimization metric. **Method D**, the proposed method, consists of four major steps: (1) generate sCT from the baseline MR image, (2) using OpenREGGUI, perform DIR between the sCT and the corresponding mCT with block matching as field computation with minimizing the SSD between the sCT and the mCT image as the optimization metric, (3) extracting the deformation field from the previous step, and (4) apply the deformation field onto the MR. In summary, in method D, the MR is coregistered with the mCT using sCT as an intermediate. **Method E** uses MIM Maestro to perform DIR based on proprietary metrics that are generated for each voxel from both MR and CT images and involves manual definition of a bounding box, in our case capturing kidneys and nearby spine, as the area of focus for registration accuracy. Although Maestro does not require defining a bounding box, we took this approach during proof‐of‐concept work using a prior version (6.6.10) to resolve the difficulties in registering bone structures and continued to use it in the work presented herein. **Method F** is a two‐step process entailing first E then refining that result using method D. The point of F is to assess the potential for the proposed sCT intermediate approach to improve upon results of a state‐of‐the‐art commercial product.

Although outside of user control of OpenREGGUI, we note that for method B (minimizing LPD), there are 6 quadrature filters, corresponding to 6 frequencies in the 3D spatial domain, that are convolved with the image to compute local phase at different scales of resolution for the LPD calculation and optimization. The displacement search is based on the analytical solution of the phase difference function between the two images among all frequencies and spatial directions. For methods C and D, OpenREGGUI uses block matching for field computation at each scale for 27 adjacent blocks.^16^

### 2.5 Registration performance evaluation

Twenty‐five sets of abdominopelvic MR OP and paired CT data are used in order to evaluate the performance of registration. Quantitative evaluations are performed using all six registration methods (A–F), and qualitative evaluation is performed for registration methods A, D, E, and F to avoid unnecessary physician effort as explained in the later section. The baseline MR images, having only rigid registration, are included as a control. Therefore, a total of 150 image volume pairs are evaluated.

#### 2.5.1 Quantitative evaluation

Quantitative evaluation provides an objective numerical value of similarity between two images to assess the registration accuracy. Specifically, the MI and LPD per voxel are calculated between the MR and the CT volumes for all six methods. Although the effect of the quality of the sCT in image registration is not the scope of this study, we report the mean of SSD and mean absolute HU difference (MAD) per voxel inside the body between the sCT and CT for all methods as a measure of registration accuracy. After the registration, the final deformation field, generated from the baseline MR image, is extracted and applied onto the sCT for calculating the SSD and MAD. In the case of methods D and F, which use the sCT as an intermediate, the final deformed sCT is readily available to calculate these metrics.

Besides the global metrics (SSD, MAD, LPD, and MI), there are quantitative evaluations based on landmark analysis. Bilateral kidneys, femoral heads, bladder, and rectum are picked as the landmarks. The kidneys are picked as they have distinct edges in both MR and CT images, and the others are picked as they are the common organs‐at‐risk for radiation treatment in the abdominopelvic region. Regions of interest (ROIs) are drawn by manually tracing contours from each subject's MR OP images and CT images. The modified Hausdorff distance (MHD) is calculated^50^:

(1) $$\text{MHD}\left(A,B\right)=\max\left\{\frac{1}{N_{a}}\sum_{x\in A}\;\text{in}f_{y\varepsilon B}d\left(x,y\right),\;\frac{1}{N_{b}}\sum_{x\in B}\;\text{in}f_{x\varepsilon A}d\left(x,y\right)\right\}$$

where A represents the contour generated from the CT image, and B represents the contour generated from the corresponding MR OP image. $\mathrm{in}f_{y\in B}d\left(x,y\right)$ represents the infimum and numerically equals the “minimum” distance from 3D position of point x in A to 3D position point y in B. *N_a_* and *N_b_* are the numbers of points in A and B. The Dice Similarity Index (DSI) is calculated:

(2) $$\mathrm{DSI}\left(A,B\right)=\frac{2\left|A\cap B\right|}{\left(\left|A\right|+\left|B\right|\right)}$$

The Jacobian determinant (JD) of the deformation field measures the change in volume for each voxel.^51^ JD values >1 correspond to a local expansion and <1 to shrinkage. A JD of less than or equal to zero indicates that there may be an error or limitation in the registration algorithm.^52^ JD values provide insights including: (1) Whether the image volume undergoes a change in total volume or not, (2) which voxels undergo a large change, and (3) if there is any limitation or error in the registration algorithm. The mean JD value of 1 is desirable as the deformable registration should not change the absolute volume of a subject. Having some voxels with JD much different than 1 supports that a rigid registration alone is not sufficient.

#### 2.5.2 Qualitative evaluation

While quantitative evaluation provides an objective measure of registration that an algorithm can utilize for optimization, it may not necessarily indicate clinical usefulness. As reviewer assessment of registration accuracy from all subjects and all methods is not practicable, we used the quantitative evaluations to eliminate the subset of methods that were clearly inferior from qualitative evaluation. (As will be shown in results, B and C were excluded.) Specifically, Methods D, E, and F, were evaluated by reviewers to clearly identify clinical usefulness of each method. Method A (rigid registration) is included as a reference (control) method. Consequently, there were 100 registered, de‐identified image pairs for 25 subjects for each of the four registration methods to be evaluated. Four clinicians were recruited to score the quality of the MR to CT registration using a Likert scale ranging from 1 to 5. Scores are assigned as:

1. Not usable; major misregistration and completely unusable
2. Bad; substantial misregistration and prefer not to use
3. Good; good enough to use, but still needs manual adjustment
4. Readable; some misalignment, but not significant such that manual adjustment is not needed
5. Perfect; no need to put effort into interpreting and distinguishing the organs from fusion images

The four board‐certified physicians recruited to help with the final evaluation of the presented work include two attending nuclear medicine physicians, a radiologist in a nuclear radiology fellowship program, and an attending cardiothoracic radiologist. MIM Maestro, a tool familiar to the clinicians, is used for image review.

### 2.6 Processing times

Synthetic CT generation and image registration are performed using a desktop computer equipped with an Intel i5‐4590 3.3 GHz CPU with 12 GB of memory and Windows 10 OS. As the number of axial slices in each volume 0 varies in different subjects, for standardization, the computer processing time for volume is divided by the number of slices in the volume, which is supported by scatter‐plot analysis showing that the computational time tends to scale linearly with the number of slices. Summary statistics of mean and standard deviation are calculated.

### 2.7 Statistical analysis

For all quantitative evaluation metrics, statistical significance is assessed using the *p*‐values calculated using one‐tailed, paired *t*‐tests. The *t*‐test is constructed to test against the alternative hypothesis that the mean of the reference method (registration accuracy) is better than that of the comparison method. The null hypothesis is rejected, and an alternative hypothesis is accepted if *p*‐value is less than 0.05. Specifically, better registration accuracy is obtained with higher values of MI and DSI and with lower values of SSD, MAD, MHD, and LPD. Therefore, right‐tailed tests are conducted for MI and DSI, and left‐tailed tests are conducted for SSD, MAD, MHD and LPD.

For qualitative evaluation of the Likert scores, which are ordinal data, statistical significance is assessed using the *p*‐values calculated using one‐tailed, Mann‐Whitney *U* test. The Mann‐Whitney *U* test is constructed to test against the alternative hypothesis that the sum of ranks of the Likert scores of the reference method is higher (better registration accuracy) than that of the comparison method. The proportion of the data sets that have Likert scores that exceed a specified threshold is calculated, and the ability of one method to achieve a statistically significantly higher proportion than another is evaluated using a chi‐squared test. The upper‐tailed test is constructed with the alternative hypothesis that the probability of the reference method having a higher proportion than that of the comparison method. For both statistics, the null hypothesis is rejected, and an alternative hypothesis is accepted if *p*‐value is less than 0.05. Likert scores given by each reviewer are analyzed to estimate inter‐reviewer variability, and composite Likert scores from all reviewers are analyzed to compare comprehensive registration performance.

## 3 RESULTS

### 3.1 Optimization metrics (SSD, MI, LPD) evaluation

**FIGURE 2:**
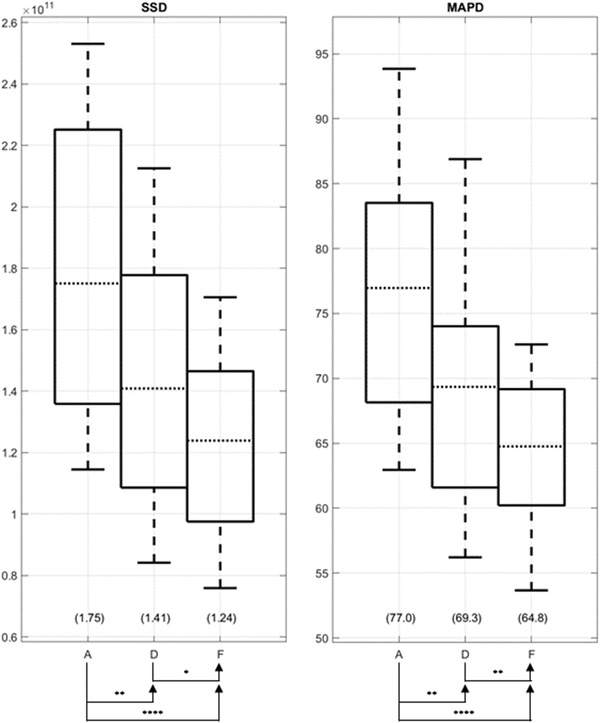
Sum of squared differences (SSDs) and mean absolute differences (MADs) comparisons of the baseline volumes (method A) and those deformably registered using different methods (methods D and E). Baseline refers to images that were aligned using rigid registration, and which served as the input to deformable registration. Generally, low SSD and MAD are desired for registrations that use sCT as an intermediate. The proposed method achieves a lower SSD and MAD than method A, and method F achieves the lowest values for both metrics. For this and subsequent box‐plots, the arrow heads indicate that the particular method achieved a better mean (or sum of ranks for qualitative evaluation) than the comparison method at the arrow tail. *p*‐Values for comparisons are shown with different levels of statistical significance represented by the number of asterisks; more asterisks represents higher statistical significance while “ns” represents not significant. The dotted horizontal lines indicate the mean values (or median for qualitative evaluation). The box heights indicate interquartile range (IQR) in between the 25th to 75th percentile (Q1 and Q3, respectively). The whiskers indicate the minimum and maximum values not considered outliers, where the outlier is determined if the value exceeds 1.5 times the IQR from Q1 and Q3. The outliers are plotted with “o.” Note that the y‐axis does not start at zero but is scaled to capture the absolute minimum and maximum values of each evaluation metric. The values in the parenthesis below the boxplots indicate the mean values.

Using the proposed method, sCTs are generated and registered with the mCT images using SSD minimization. Five examples of such sCT images are shown in Figure S1. We used the HU difference between the registered sCT and CT as one measure of the registration accuracy; a superior registration should minimize the HU difference recognizing that the differences would not reach zero as they are also affected by the accuracy of the sCT per se. Specifically, Figure S2 shows the SSD and voxel‐wise MAD, that is perhaps a more intuitive measure, of all methods. When ordered by the SSD and MAD values, the methods are F < D < A < C < B < E from lowest to highest, or from best to worst registration, and numeric values of statistical significance between the successive methods in the ordered list (e.g., F vs. D, D vs. A, etc.) are listed in Tables S1 and S2 for SSD and MAD, respectively. Considering the methods that optimize SSD (D and F), Figure 2 plots SSD and MAD compared to those of the rigid registration method A as reference, with statistical significance versus method A annotated. Indeed, as expected, D and F significantly reduce SSD and MAD as well. It should be noted that the MADs are calculated excluding the background air outside the body to avoid diluting the result by many pixels that are perfectly matched. The somewhat large MAD is mainly attributed to a small subset of the voxels which are (1) the outliers that result from misclassifications of bone and air inside the body, particularly in the lungs and around the spinal canal, and (2) the outliers result from anomalies near the superior and inferior ends of the field of view (FOV), wherein some spatial locations have data available from one modality but not the other. Both of these outliers may often cause some voxel differences to exceed 1000 HU. Despite the outliers, the methods that use sCT as an intermediate (D and F) seem to be robust with regard to the presence of the outliers noting that we did not omit these outliers in the registration process.

**FIGURE 3:**
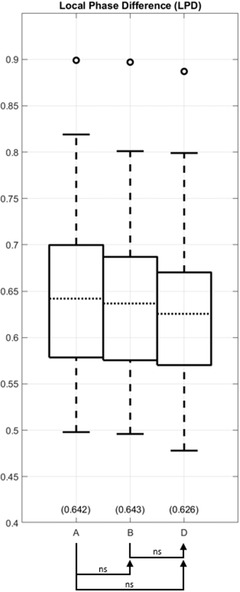
Local phase difference (LPD) comparison of the baseline volumes (method A) and those deformably registered using different methods (methods B and D). The arrow head annotations indicate that the particular method achieved a lower (better) LPD value than the comparison method at the arrow tail. The proposed method achieves a lower LPD than method B, which directly optimizes LPD.

**FIGURE 4:**
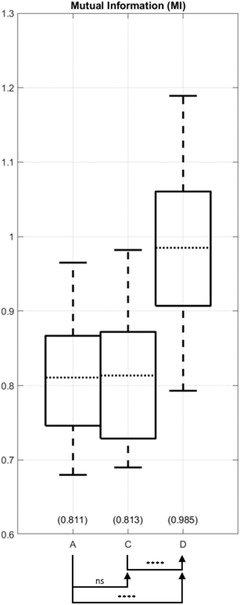
Mutual information (MI) comparison of the baseline volumes (method A) and those deformably registered using different methods (methods C and D). The arrow head annotations indicate that the particular method achieved a higher (better) MI value than the comparison method at the arrow tail. The proposed method achieves a higher MI than method C, which directly optimizes MI.

As another quantitative assessment of registration methods, the LPD and MI values are calculated for each of the six registration results (including, even the methods that do not minimize LPD or maximize MI). Full results of LPD and MI values are plotted in Figures S3 and S4, respectively. When ordered by mean LPD, the methods are E < D < B < F < A < C, from lowest to highest values, or from best to worst registration accuracy. Statistical analysis comparing successive methods in this ordered list did not achieve significance as detailed in Table S3. However, method E achieves statistically significant lower LPD than method B (*p* = 0.042). Figure 3 plots LPD for method A the reference rigid registration, method B that optimizes LPD, and D our proposed method. Our proposed method shows lower LPD than method B, although the difference did not achieve statistical significance. Turning to MI as the metric, the methods ranked E > F > D > B > C > A from highest to lowest values, or from best to worst registration accuracy. Statistical analysis comparing successive methods in the ordered list are presented in Table S4. Considering A, B, and C as one group and D, E, and F as the other group, we successively compared a member of the second group with members of the first group for a total of nine comparison pairs: (D vs. [A, B, or C]; E vs. [A, B, or C]; and F vs. [A, B, or C]). All nine comparison pairs found D, E, and F are superior to A, B, and C (*p* < 0.0001). Figure 4 plots MI for method A the reference rigid registration, method C that optimizes MI, and D our proposed method. Method C fails to achieve a statistically significant difference against method A (*p* = 0.44), while method D achieved a significantly higher MI than both methods A and C.

#### 3.1.1 Landmark error evaluation

Differences in the registration accuracy of the methods based on LPD versus MI indicates that these metrics alone are not sufficient to unambiguously make a conclusion about which method should be preferred; hence we also explored landmark error evaluation. Registration accuracy is scored based on the spatial correspondence of anatomic structures as seen in CT and MR. Spatial correspondence is quantified using the MHD and DSI to assess ROI contours drawn on each subject's mCT image and MR OP images after registration.

**FIGURE 5:**
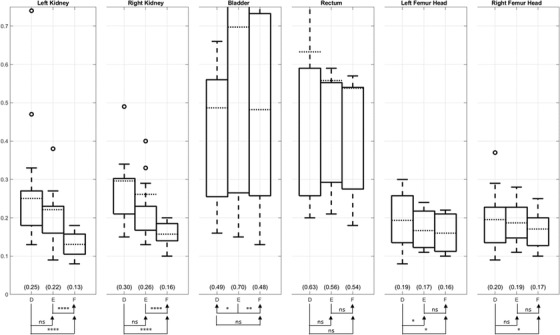
Modified HD (MHD) values comparison of region of interest (ROI) between the registered magnetic resonance (MR) opposed‐phase (OP) images from methods D, E, and F and the measured computed tomography (CT) images. Generally, lower MHD is desired for registration. Method E shows relatively better MHD than method D, and method F shows relatively better MHD than method E.

Full results for landmark evaluation using MHD are shown in the supplementary materials Figure S5 and Table S5. Surprisingly, method B degrades the MHD after DIR for all ROIs, meaning method B performs even worse than rigid registration (method A). Generally, when ordered by mean MHD, the methods are F < E < D < A < C < B, from lowest to highest values, or from best to worst registration accuracy, and numeric values of statistical significance between the successive methods in the ordered list are listed in Table S5. Considering A, B, and C as one group and D, E, and F as the other group, we successively compared a member of the second group with members of the first group for a total of 9 comparison pairs: (D vs. [A, B, or C]; E vs. [A, B, or C]; and F vs. [A, B, or C]). All nine comparison pairs found D, E, and F are superior to A, B, and C for all ROIs with statistical significance achieved for bilateral kidneys and bilateral femur heads (*p* < 0.0001) but not for bladder and rectum (*p* < 0.25). Focusing on the best‐performing methods D, E, and F, box plots analysis (Figure 5) shows that: (1) method E demonstrates lower MHD than method D but does not achieve statistical significance for most ROIs except for the left femoral head, and (2) method F demonstrates lower MHD than method E for all ROIs with statistical significance achieved except for rectum and left femur head.

**FIGURE 6:**
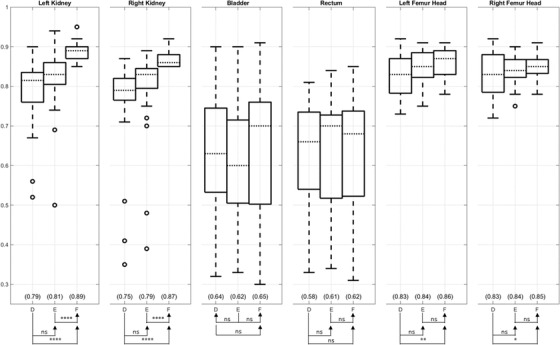
Dice similarity index (DSI) values comparison of region of interest (ROI) between the registered magnetic resonance (MR) opposed‐phase (OP) images from methods D, E, and F and the measured computed tomography (CT) images. Generally, higher DSI is desired for registration. Method E shows relatively better DSI than method D, and method F shows relatively better DSI than method E.

Full results for landmark evaluation using DSI are shown in the supplementary materials Figure S7 and Table S6. Surprisingly, methods B and C degrade the DSI after DIR for most ROIs, meaning methods B and C performs even worse than rigid registration (method A). Generally, when ordered by mean DSI, the methods are F > E > D > A > C > B, from highest to lowest values, or from best to worst registration accuracy. Statistical analysis comparing between the successive methods in the ordered list are presented in Table S6. Considering A, B, and C as one group and D, E, and F as the other group, we successively compared a member of the second group with members of the first group for a total of nine comparison pairs: (D vs. [A, B, or C]; E vs. [A, B, or C]; and F vs. [A, B, or C]). All nine comparison pairs found D, E, and F are superior to A, B, and C for all ROIs (*p* < 0.009). Focusing on the best‐performing methods, Figure 6 shows that (1) method E achieves improvement over method D but does not achieve statistical significance for any ROI, and (2) method F achieves a higher DSI mean on all ROI than method E with statistical significance on the left and right kidneys.

### 3.2 Jacobian determinant

**FIGURE 7:**
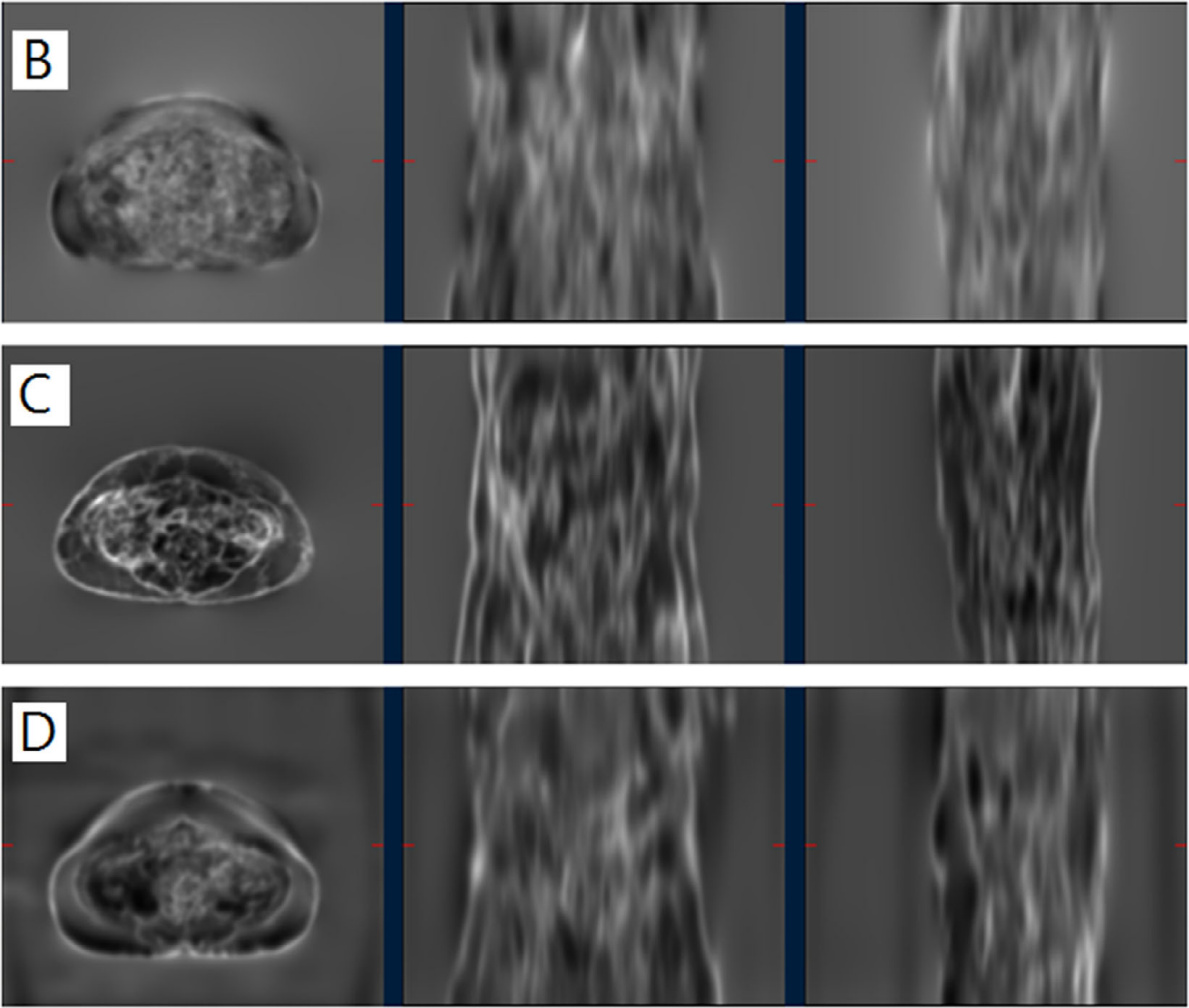
Jacobian determinant (JD) maps of the magnetic resonance (MR) opposed‐phase (OP) to computed tomography (CT) registration deformation fields of a typical subject, using the local phase difference (LPD)‐based method (B), the mutual information (MI)‐based method (C), or the proposed method (D). Note that the bright regions indicate local expansion, and the dark regions indicate shrinkage. These JD maps show the largest variation on the body edges and organ boundaries. Method B shows the largest deformation among the compared methods.

**FIGURE 8:**
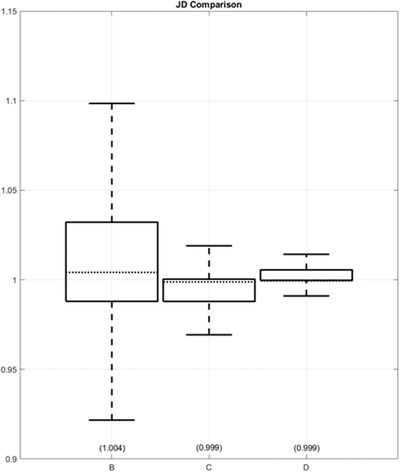
The statistical plot of Jacobian determinant (JD) values of all subjects using the three methods (B, C, and D). The boxplot shows that method B has the largest interquartile range (IQR), indicating greatest deformation at certain locations. Note that the outliers are not plotted as part of the boxplots because, due to hundreds of millions of samples, their number is so great that plotting them obstructs the visualization of the boxplot. Instead, we report that for methods B, C, and D, respectively, 1) the ranges are (0.43, 2.00), (0.72, 1.57), and (0.51, 2.44); 2) 67.3%, 84.8%, and 82.9% of JD values are between 0.95 and 1.05; and 3) 90.2%, 98.5%, and 96.8% of JD values are between 0.85 and 1.15.

**FIGURE 9:**
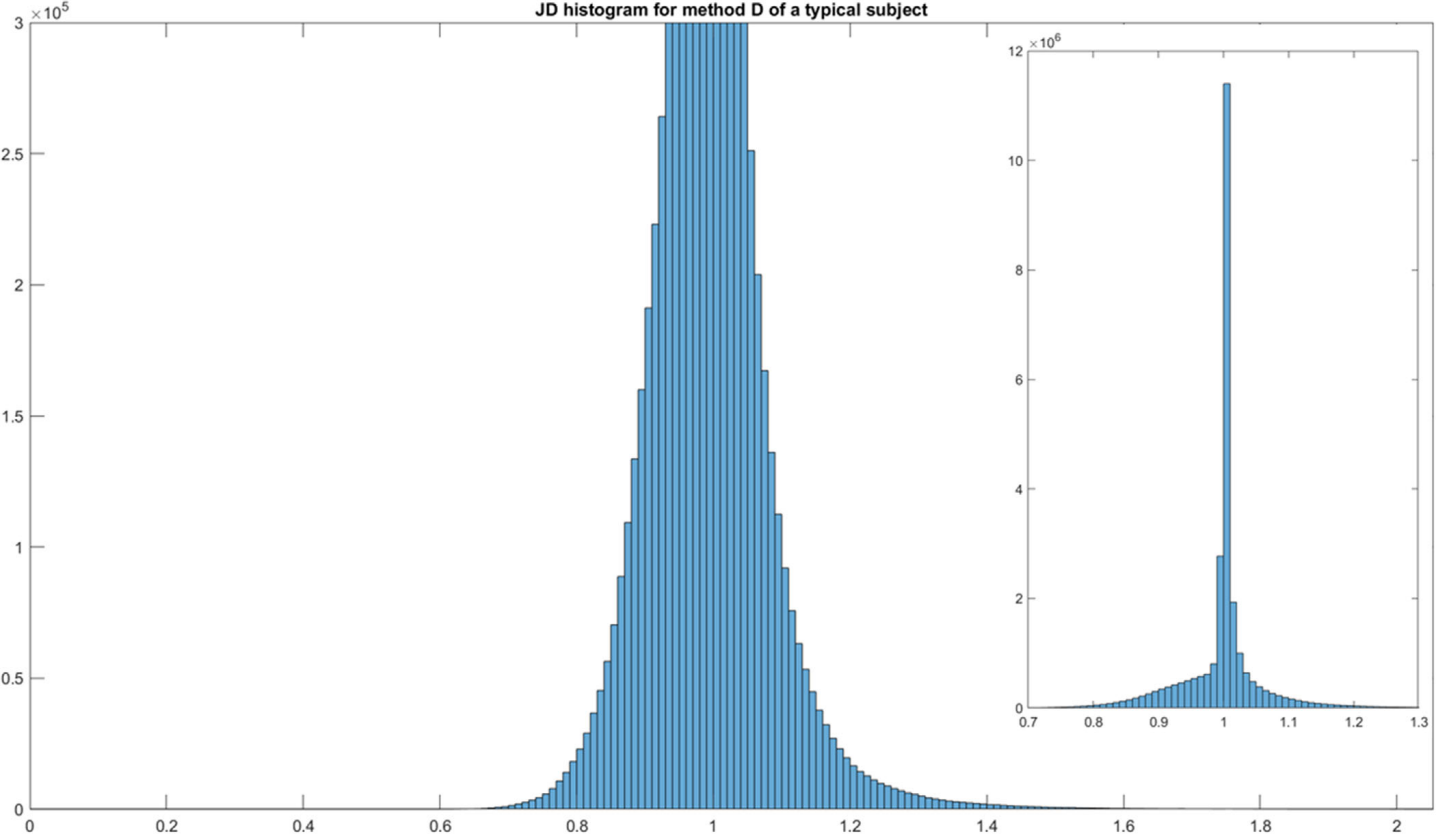
Jacobian determinant (JD) histogram of the magnetic resonance (MR) opposed‐phase (OP) to computed tomography (CT) registration deformation fields of a typical subject, using the proposed method (D). The main plot shows a histogram magnified for a shorter y‐axis (number of voxels) than the subplot on the right, which is a histogram capturing the maximum height occurring in between JD values of 1 and 1.01. The minimum JD value is approximately 0.5, and the maximum JD value is approximately 2.1, which are not distinguishable from the histogram as there are only a handful of voxels with such small and large values.

The JD measures the degree of deformation at each voxel. JD maps provide intuitive visualization of the deformation. Figure 7 shows example maps for the deformation fields of methods B, C, and D for a typical subject; maps are not available for methods A, E, and F as MIM Maestro does not make these available. Figure 8 shows a boxplot visualization of the statistical summary of the distributions of JD values across all subjects to indicate the change in volume for each voxel after the transformation. As evident in Figure 8, although the mean JD values are all close to 1 for methods B, C, and D, method B shows a larger interquartile range than the other two methods C and D, indicating larger deformations at certain voxels. Delving into the details for an example data set, Figure 9 shows a histogram of JD values for the deformation field of method D for a typical subject. There are some voxels with JD values as small as 0.5 and as large as 2, but these voxels are extremely rare as evident in the histogram noting that the inset plot shows that the JD values of most voxels are between 0.95 and 1.05. Specifically, for methods B, C, and D respectively: (1) ranges are (0.43–2.00), (0.72–1.57), and (0.51–2.44), (2) 67.3%, 84.8%, and 82.9% of JD values are between 0.95 and 1.05, and (3) 90.2%, 98.5%, and 96.8% of JD values are between 0.85 and 1.15. JD values smaller than 0.85 and larger than 1.15 mostly occur at four locations: (1) inferior boundary of lungs, (2) spinal canal, (3) intestines, and (4) medullary cavity of femur bone.

### 3.3 Qualitative evaluation

**FIGURE 10:**
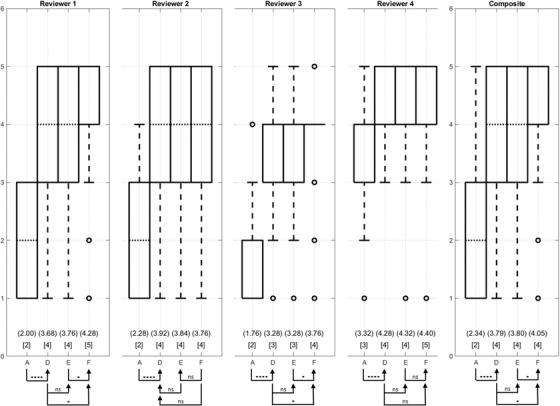
Box plots summarize the distribution of the scores by the reviewers, including the composition of all physician evaluation. The values in the square bracket below the boxplot represent the median values, which are indicated as dotted horizontal lines. The values in the parenthesis are the mean Likert scores and are provided as a simple metric that can be calculated for each method, whereas the sum of ranks must be calculated separately (pairwise) for every comparison. To evaluate potential for statistically significant differences, sum‐of‐ranks /Mann‐Whitney *U* test tests were performed. The arrow head annotations indicate that the particular method achieved a higher (better) sum of ranks than the comparison method at the arrow tail. For each reviewer, there were 100 cases to evaluate with 25 cases for each of the four methods. Generally, method F received the highest scores across all the physicians. *p*‐Values between method F, and the three other methods indicate that the performance advantages between method F versus other methods are statistically significant. Method E has a tendency to perform better than method D but fails to achieve statistical significance. Note that the reviewer 2 preferred method D, although statistical significance is not achieved.

To assess the potential for clinically relevant improvements in registration, reviewers reviewed the image volumes, visually scoring the registration accuracy of the reference, A, and of the best‐performing methods D, E, and F. Unsurprisingly, the specific area of concern for any individual reviewer is related to that reviewer's area of clinical practice with our different reviewers noting that the features of concern are the bone, intramuscular fat, major organ bodies, vascular, and patient‐specific pathology. Figure 10 box plots provide a visual summary of the distributions and annotations of statistical significance of the scores assigned by each of the reviewers and a composite of all reviewers. A tally of the number of images achieving each score, aggregated across all reviewers, is presented in Table 2. Considering a composite of all reviewers’ Likert scores, the relative registration performance is in the order of methods F > E > D > A, from best to worst registration accuracy evaluated by sum of ranks. Statistical analysis comparing successive methods in the ordered list is presented in Table S7. F is better than E (*p* = 0.048), and D is better than A (*p* = 2.54 × 10^–15^); however the difference between D and E fails to achieve statistical significance (*p* = 0.35). That is, choice of method D versus E for registration is not expected to be clinically relevant.

**TABLE 2:** Number of cases for each Likert score for each method from all physician evaluation

	A	D	E	F
Score 1	29	4	8	5
Score 2	27	9	6	7
Score 3	29	20	18	10
Score 4	11	38	34	34
Score 5	4	29	34	44

*Note*: Score >3 indicates a sufficient registration accuracy is achieved that manual refinement is not needed. Score 5 indicates a perfect registration of clinically relevant organs.

To address the question of the ability of a fully‐automated DIR method to achieve sufficient registration accuracy that manual refinement is not needed, we calculated the proportion of cases, which had Likert score >3 for each method. The methods, ordered by the proportion, are F > E > D > A, from highest to lowest proportions. While methods D and E show similar proportions of scans not requiring manual refinement (Likert score > 3) (*p* = 0.88), method F tends to a higher proportion than method E (*p* = 0.11) and method D (*p* = 0.08).

### 3.4 Processing time

In order to gauge the practicality of using the various fully automatic registration methods, the processing times are provided in Table 3. Note that methods D and F have two time‐consuming steps:

**TABLE 3:** The mean processing time per (512 × 512) slice for each method

Steps	A	B	C	D	E	F
sCT generation				2.3 s		2.3 s
Registration	0.1 s	8.2 s	183.2 s	5 s	2 s	5.6 s
Total time	0.1 s	8.2 s	183.2 s	7.3 s	2 s	7.9 s
Total time for the volume	8 s	12 min	4.5 h	7.4 min	2.9 min	8.3 min

*Note*: The last row is the mean processing time for the volume. Methods D and F have an additional time‐consuming step of generating sCT from the MR volume. The registration processing time for method F includes both the DIR from MIM Maestro (method E) and proposed method from OpenREGGUI (method D).

(1) the mean processing time for generating a sCT volume from the MR volume and (2) the mean processing time taken to register MR to CT data. With an exception of method B and C, all methods show reasonable processing times to be implemented for a fully automatic registration process with processing times less than 9 min per volume. The registration processing time for method F includes both the DIR from MIM Maestro (method E) and proposed method from OpenREGGUI (method D). Note that it exhibits shorter processing time than adding those of methods D and E, presumably because registering sCT to the mCT is faster than method D as the sCT generated from method E, and used for method F, is already registered to the mCT via MR‐CT coregistration from method E.

## 4 DISCUSSION

Herein a method of transforming the MR to CT image registration problem into a same‐modality registration task using sCT as an intermediate is evaluated. In particular, and unlike prior work,^30^, ^33^ the proposed method uses SSD as the similarity metric and overcomes some of the robustness challenges of direct multimodality registration based on optimizing MI or LPD. In summary, we recommend method D as clinically practicable and valuable for registering MR and CT with its fully automatic workflow, particularly when Maestro is not available as might be the case in an academic research environment. One of the greatest potential benefits this study can offer for radiation oncology treatment planning is transferring the contours drawn on MR images to CT images for better confidence in localization of highly deformable organs (such as bladder and rectum) and reproductive organs (such as prostate, uterus and cervix) than when using CT standalone, which lacks contrast thus limiting identification of critical organs. Therefore, future studies may include clinical validation of the transferred contours. Another use of our proposed methodology is to create a virtual PET/MR study by accurate coregistration of MR with a CT image volume that is acquired as part of a PET/CT examination. This provides PET/MR functionality in the majority of sites that do not have such physical scanners.

### 4.1 General ranking order of methods

Considering clinically relevant measures, the general order of methods in terms of registration accuracy is F > E > D > A > C > B, from best to worst, with method D being, in most cases, the preferred method with its fully automatic workflow. Methods D, E, and F, as a group, achieve clinically relevant registration accuracy that is significantly better than that of conventional methods A, B, and C as can be seen in Figures S5–S7. Figure S7 illustrates this using a checkerboard display presentation that, in hard‐copy format, better depicts the registration performance than Figure S8, which illustrates a snapshot from the interactive clinical display tool that reviewers used to evaluate the paired MR‐CT image volumes. In particular, the checkerboard display enhances the visualization of the discontinuities of anatomical structures between the mCT and Dixon OP images and confirms that discontinuities are more prevalent in methods A, B, and C than in methods D, E, and F.

Evaluation results suggest that our proposed method D can be an alternative for MIM Maestro DIR (method E) particularly when Maestro is not available as might be the case in an academic research environment. On the other hand, the FDA clearance, user friendliness, and user support may make Maestro preferable for routine clinical use. Indeed, neither was clearly superior to the other. In particular, the difference in rank sum of Likert scores between methods D and E is not statistically significant (*p* = 0.35), and the difference in the number of cases not requiring additional manual adjustment (Likert score > 3) is also not statistically significant (*p* = 0.88). In fact, this high *p*‐value suggests that methods D and E are trending toward being indistinguishable. A particular advantage of the proposed method D is that it has potentially better reproducibility due to its fully automatic operation compared to MIM Maestro DIR (method E), as we used it, which involves manually choosing a bounding box. Maestro does not require selecting a bounding box, but our group implemented it during proof‐of‐concept work using a prior version of the software to resolve the difficulties in registering bone structures, and continued to use it in the work presented herein. On the other hand, method F, using our method to refine the results achieved by MIM Maestro, did achieve a superior result by some metrics. The difference in rank sum of Likert scores between methods F versus D is significant (*p* = 0.016), but the difference in utility of fully automatic DIR based on the number of cases that achieved Likert score > 3 did not achieve statistical significance (*p* = 0.08). Moreover, the checkerboard visualization, Figure S7, does not reveal any major differences in terms of spatial discontinuities between methods D, E, and F. Considering this and that this two‐step approach of F and entailing both clinical and research software might be impracticable for routine clinical practice in its current implementation. A possibility for future work would be to achieve a clinically practical streamlined workflow by addressed by implementing method D in MIM Extensions.

### 4.2 Relative utility of metrics for computerized optimization

Automated registration requires an objective measure of registration goodness that can be optimized, and, for this purpose, we considered LPD, MI, and SSD. Although MI‐based registration may outperform LPD‐based registration, as implemented herein, both metrics seem to be particularly more challenging to optimize and less robust than SSD. The rank of methods when ordered by LPD (E > D > B > F > A > C) is inconsistent with the general rank order of methods in terms of clinically relevant registration accuracy (F > E > D > A > C > B), suggesting limited robustness of LPD. Despite the inconsistency, we do not attribute it to an intrinsic flaw of LPD as a metric because the result is consistent with the general findings with regard to clinical registration accuracy in that: (1) method E shows statistically significantly lower (better) LPD than methods A, B, and C, and (2) the difference in LPD between methods D and E does not achieve statistical significance. Moreover, evidence that the LPD optimization is implemented correctly is that method B, which directly optimizes LPD, achieves lower (better) LPD than the initial guess in the optimization (rigid registration, i.e., method A), so the fact that method E achieves statistically significantly lower (better) LPD than method B (*p* = 0.042) suggests that LPD is difficult to optimize. Indeed, LPD is known to lack a common set of filters that generally yield a global optimum across various multidimensional images of different anatomical regions, implying a possibility of the existence of different sets of filters that can reflect clinically relevant registration accuracy better than the filters implemented in this study.^29^, ^53^ Therefore, future studies may include a systematic search for a common set of filters that achieves the best clinically relevant registration accuracy.

In considering MI as a metric, the rank of methods when ordered by MI (E > F > D > B > C > A) is more consistent with that of clinically relevant registration accuracy rank order of methods (F > E > D > A > C > B) than when ordered by LPD. Also, the MI metric finds that methods D, E, and F, as a group, achieve statistically significantly higher (better) MI than methods A, B, and C, as a group, as is the case with the clinically relevant registration accuracy (Likert scores), but not with LPD as a metric. Although MI, as metric, is more robust than LPD, MI too can be challenging for automated computer optimization. Evidence that the MI optimization is implemented correctly is found in method C, which directly optimizes MI, improves the MI from the initial guess (rigid registration, i.e., method A), so the fact that method D, which optimizes SSD, often achieves the highest (best) MI value suggests that MI is difficult to optimize. Our experience that MI is difficult to optimize concurs with that of others who report that the MI objective function has a relatively narrow, peaked region around the optimal point with a flat area beyond the neighborhood.^34^, ^35^ Another limitation of MI is that it does not achieve statistical significance in identifying the difference between methods D and F in contrast to the clinically relevant measures (Likert score, MHD, and DSI) which do.

In contrast to evidence of limited robustness of LPD and MI, SSD, as a metric, seems to be more robust and reliable as ranking by SSD reflects clinically relevant registration accuracy better than ranking by LPD or MI. The rank of methods when ordered by SSD (F > D > A > C > B > E) is, with the exception of E, consistent with the clinically relevant rank order of registration accuracy (F > E > D > A > C > B). Method E achieves the highest (worst) SSD, but we attribute this to the vagaries of how MIM Maestro handles the deformation field toward the axial ends of the FOV ‐ repositioning anatomy that is outside of the FOV of one image volume set but not of the other. In particular, noting that we calculate SSD for method E by extracting the resultant deformation field from MIM Maestro and apply it to the initial sCT, the high SSD is ameliorated when end planes are omitted from the SSD calculation. Thus, for these reasons, we find SSD to be the superior registration metric. Also, it seems to be robust with respect to imperfections in the sCT accuracy, and thus we did not have to resort to a ultrashort echo time or zero echo time pulse sequence to improve the discrimination between bone and air for sCT generation; the commonly used Dixon pulse sequence was sufficient. Another advantage is that SSD is much faster to optimize than LPD and MI as presented in Table 3, further supporting that SSD is clinically more practical than LPD and MI. This, coupled with the fully automated processing of method D means that the registration can be seemingly instantaneous from the physicians’ perspective as it is feasible to perform it after image acquisition and before they are ready to read the images. It is worthwhile to note that, of the two SSD‐based methods, F achieves better SSD than method D (*p* = 0.0037). Thus, even with SSD, there is some challenge in optimizing SSD and finding the global optimum given that the only difference is that D and F start with different initial guesses. Therefore, future studies may consider strategies to find the global minimum of the SSD.

### 4.3 Challenges of DIR in abdominopelvic region

The abdominopelvic region is a particularly challenging anatomic section to perform DIR because organs are highly deformable. Contours, assessed using MHD and DSI, are consistent with physician review, but even with methods D, E, and F, the mean MHD can exceed 4 mm and the DSI similarity be less than 0.7 for bladder and rectum with large standard deviations across subjects. However, this does not represent intrinsic limitations of methods D, E, and F as MR and CT are acquired on different scanners—there is no combined MR‐CT scanner, which necessitates patient repositioning and time gaps, which, in our case, were on the order of 30 min. Consequently, the bladder size and shape can be different across subjects due to filling and the mobility of adjacent organs. For example, the rectum is a relatively deformable organ, and its volume can be affected by bladder filling and bowel gas, so a large MHD and a small DSI in these areas are not surprising. As presented in Figure 7, deformations mainly occur on the body edges and organ boundaries that are affected by breathing motions and deformable nature of organs, further illustrating the challenges of abdominopelvic DIR.

## 5 CONCLUSION

This work proposes the methodology of using a sCT as an intermediate for the abdominopelvic MR to CT registration. This maps the multimodality registration problem to the simpler same modality registration that can use SSD as the metric to minimize. The deformation field is extracted from the sCT to (measured) CT registration and then applied to the MR data to register it with CT. Both quantitative and qualitative results show: (1) improved robustness and clinically relevant registration accuracy of our proposed method compared with the traditional rigid, LPD‐ and MI‐based registration, (2) in a research environment our proposed method can provide a DIR that performs comparably to that in a commercial software, (3) our proposed method may be of interest for implementation in a commercial software to support the clinical workflow.

## CONFLICT OF INTEREST

The authors declare that there is no conflict of interest that could be perceived as prejudicing the impartiality of the research reported.

## AUTHOR CONTRIBUTIONS

Bryan J. Traughber and Raymond F. Muzic Jr. conceived of the presented method. Raymond F. Muzic Jr., Pengjiang Qian, Feifei Zhou, Jin Uk Heo, and Jiamin Zheng developed the theory and performed the computations. Feifei Zhou and Jin Uk Heo designed the experiments and wrote the manuscript with support from Bryan J. Traughber and Raymond F. Muzic, Jr. Jiamin Zheng, Xin Song, and Pengjiang Qian supported the sCT generation using a previously published method. Feifei Zhou and Jin Uk Heo performed image registration, quantitative evaluations, statistical analysis, and preparation of results. Melanie S. Traughber, Jung‐Wen Kuo, and Rose Al Helo contributed to the data collection and preprocessing. Atallah Baydoun and Christian B. Langmack guided the anatomical contouring and qualitative evaluation. Aaron Nelson and Alexandria Kruzer provided details about MIM Maestro. Norbert Avril, Amit Gupta, Robert Jones, Daniel DeVincent, Harold Hunt, Navid Faraji, Michael Z. Kharouta, Arash Kardan, and David Bitonte are the physicians who contributed to the design and performed the qualitative image analysis. Norbert Avril, Min Yao, Jennifer Dorth, John Nakayama, Steven E. Waggoner, Tithi Biswas, Eleanor Harris, and S. Kate Sandstrom are the physicians who provided the patient data. Cheryl Thompson oversaw the statistical analysis. Raymond F. Muzic Jr., Robert Jones, and Pengjiang Qian are the PIs for grants that support this study. Robert Jones is the PI of the IRB protocol. All listed authors proofread and approved of the manuscript.

## ETHICS STATEMENT

The subject recruitment, data collection, and management are in compliance with protocols reviewed and approved by the University Hospitals Cleveland Medical Center Institutional Review Board.

## Supporting information

Supporting Information

Figures

Tables
