# Supplementary material for: Abdominopelvic MR to CT registration using a synthetic CT intermediate
Source: J Appl Clin Med Phys. 2022 Aug 3;23(9):e13731. doi: 10.1002/acm2.13731 (PMC9512351; doi:10.1002/acm2.13731)
Supplement: Supplementary file 1 — Supporting Information [file ACM2-23-e13731-s002.docx]

Abdominopelvic MR to CT registration using

a synthetic CT intermediate

Jin Uk Heo^1,2^, Feifei Zhou^1^, Robert Jones^1,3^, Jiamin Zheng^4^, Xin Song^4^, Pengjiang Qian^4^,

Atallah Baydoun^2,5^, Melanie S Traughber^6^, Jung-Wen Kuo^1^, Rose Al Helo^3^, Cheryl Thompson^7^, Norbert Avril^1,3^, Daniel DeVincent^3^, Harold Hunt^3^, Amit Gupta^1,3^, Navid Faraji^1,3^, Michael Z Kharouta^8^, Arash Kardan^1,3^, David Bitonte^1,3^, Christian B. Langmack^8^, Aaron Nelson^9^, Alexandria Kruzer^9^, Min Yao^6^, Jennifer Dorth^8,10^, John Nakayama^11^, Steven E Waggoner^12^, Tithi Biswas^8,10^, Eleanor Harris^8,10^, S Kate Sandstrom^8^, Bryan J. Traughber^6^, Raymond F. Muzic, Jr.^1,2,3^

^1^ Department of Radiology, Case Western Reserve University, Cleveland, Ohio 44106, USA

^2^ Department of Biomedical Engineering, Case Western Reserve University, Cleveland, Ohio 44106, USA

^3^ Department of Radiology, University Hospitals Cleveland Medical Center, Cleveland, Ohio 44106, USA

^4^ School of Artificial Intelligence and Computer Science, Jiangnan University, Wuxi, Jiangsu 214122, China

^5^ Department of Internal Medicine, Louis Stokes Cleveland VA Medical Center, Cleveland, OH 44106, USA

^6^ Department of Radiation Oncology, Penn State University, Hershey, PA 17033, USA

^7^ Department of Public Health Sciences, Penn State College of Medicine, Hershey, PA 17033, USA

^8^ Department of Radiation Oncology, University Hospitals Cleveland Medical Center, Cleveland, OH 44106, USA

^9^ MIM Software Inc., Cleveland, Ohio 44122, USA

^10^ Department of Radiation Oncology, Case Western Reserve University, Cleveland, OH 44106, USA

^11^ Department of Obstetrics and Gynecology, Allegheny Health Network, Pittsburgh, PA 15212, USA

^12^ Department of Obstetrics and Gynecology, Cleveland Clinic, Cleveland, OH 44195, USA

Corresponding author: Raymond F. Muzic, Jr, (e-mail: [raymond.muzic@case.edu](mailto:raymond.muzic@case.edu)).

**Author Contribution Statement**

Bryan J. Traughber and Raymond F. Muzic, Jr. conceived of the presented method.

Raymond F. Muzic Jr., Pengjiang Qian, Feifei Zhou, Jin Uk Heo and Jiamin Zheng developed the theory and performed the computations.

Feifei Zhou and Jin Uk Heo designed the experiments and wrote the manuscript with support from Bryan J. Traughber and Raymond F. Muzic, Jr..

Jiamin Zheng, Xin Song, and Pengjiang Qian supported the sCT generation using a previously published method.

Feifei Zhou and Jin Uk Heo performed image registration, quantitative evaluations, statistical analysis, and preparation of results.

Melanie S Traughber, Jung-Wen Kuo and Rose Al Helo contributed to the data collection and preprocessing.

Atallah Baydoun and Christian B. Langmack guided the anatomical contouring and qualitative evaluation.

Aaron Nelson and Alexandria Kruzer provided details about MIM Maestro.

Norbert Avril, Amit Gupta, Robert Jones, Daniel DeVincent, Harold Hunt, Navid Faraji, Michael Z Kharouta, Arash Kardan and David Bitonte are the physicians who contributed to the design and performed the qualitative image analysis.

Norbert Avril, Min Yao, Jennifer Dorth, John Nakayama, Steven E Waggoner, Tithi Biswas, Eleanor Harris, S Kate Sandstrom are the physicians who provided the patient data

Cheryl Thompson oversaw the statistical analysis.

Raymond F. Muzic, Jr., Robert Jones and Pengjiang Qian are the PIs for grants that support this study. Robert Jones is the PI of the IRB protocol.

All listed authors proofread and approved of the manuscript.

**Acknowledgements**

We thank Patrick Wojtylak BS, MSHA, CNMT, Adina Crisnan BS, CNMT, Megan Fontanez BS, CNMT, Adam Gigliotti BS, CNMT, and Janine M Tessean BS, CNMT for assistance in data collection and review. We thank Victoria Uram MPH, Jennifer Sposato R.T. (R )(CT), Melissa Baker MSHS for regulatory support. This work was supported by the National Cancer Institute of the National Institutes of Health (grant number R01CA196687, PI: Muzic) and by University Hospitals Minority Faculty Development Award (PI: Jones). This work was also supported in part by the National Natural Science Foundation of China under Grants 61772241 and 61702225, by the Natural Science Foundation of Jiangsu Province under Grant BK20160187, by the Fundamental Research Funds for the Central Universities under Grant JUSRP51614A, by 2016 Qinglan Project of Jiangsu Province, by 2016 Six Talent Peaks Project of Jiangsu Province, and by the Science and Technology Demonstration Project of Social Development of Wuxi under Grant WX18IVJN002. The content is solely the responsibility of the authors and does not necessarily represent the official views of the funding agencies.

**Ethical Approval Statement**

The subject recruitment, data collection, and management are in compliance with protocols reviewed and approved by the University Hospitals Cleveland Medical Center Institutional Review Board.

- *Above ethical approval statement is redacted in the main body of the manuscript to adhere to the double-blinding review process of the JACMP.*
