# Supplementary material for: Abdominopelvic MR to CT registration using a synthetic CT intermediate
Source: J Appl Clin Med Phys. 2022 Aug 3;23(9):e13731. doi: 10.1002/acm2.13731 (PMC9512351; doi:10.1002/acm2.13731)
Supplement: Supplementary file 2 — Figures [file ACM2-23-e13731-s003.docx]

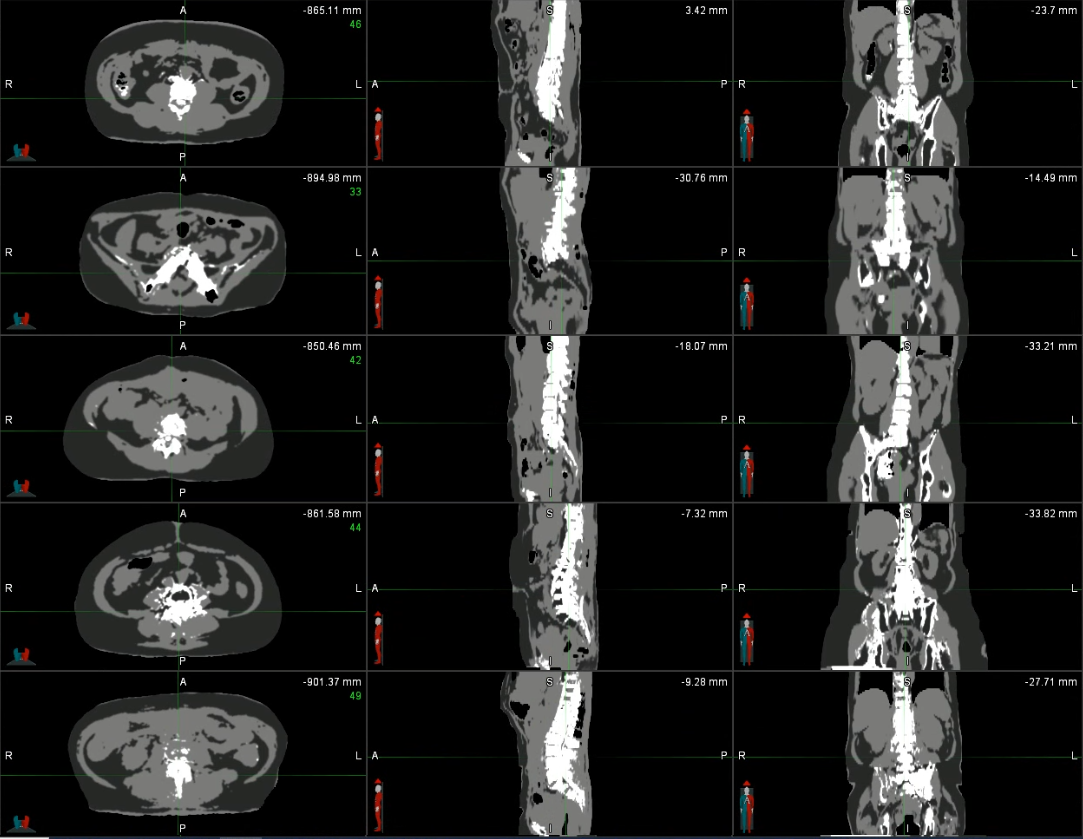


**Fig. S1.** This figure provides five example sCT images as a reference for typical subjects.


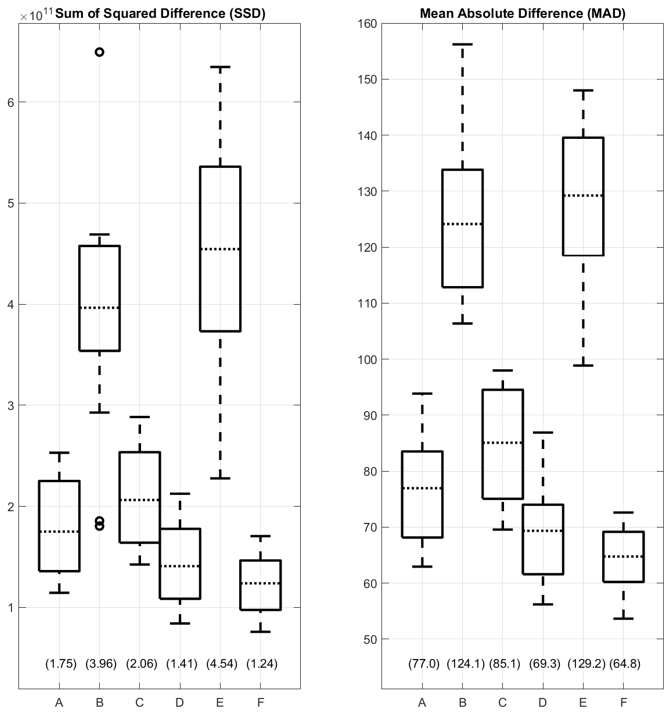


**Fig. S2.** Sum of Squared Differences (SSD) and Mean Absolute Differences (MAD) comparisons of all methods. Generally, low SSD and MAD are desired. Method F achieves the lowest values for both metrics while the proposed method achieves the second lowest SSD and MAD.


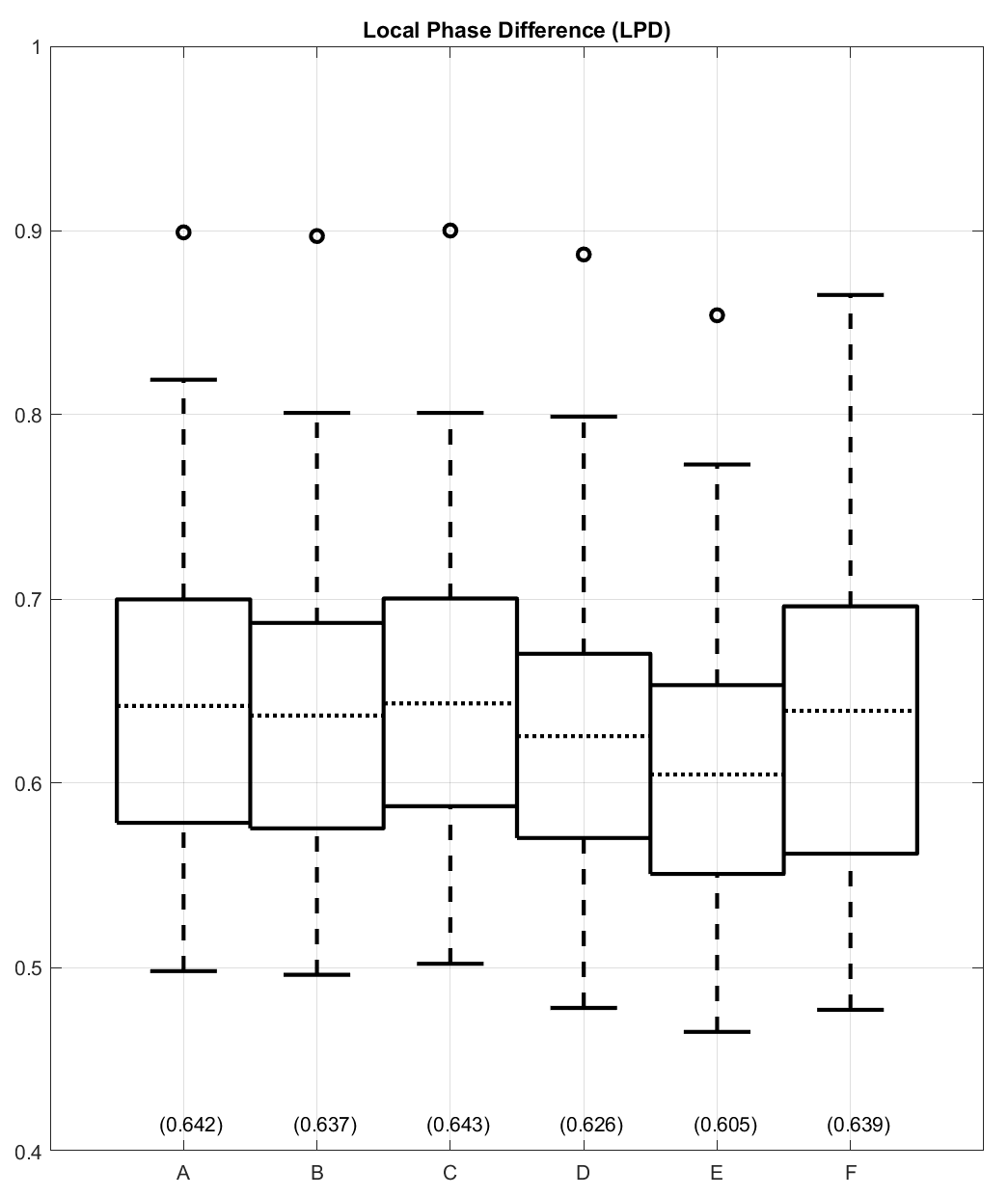


**Fig. S3.** Local Phase Difference (LPD) comparison of all six methods. Method E achieves the lowest LPD.


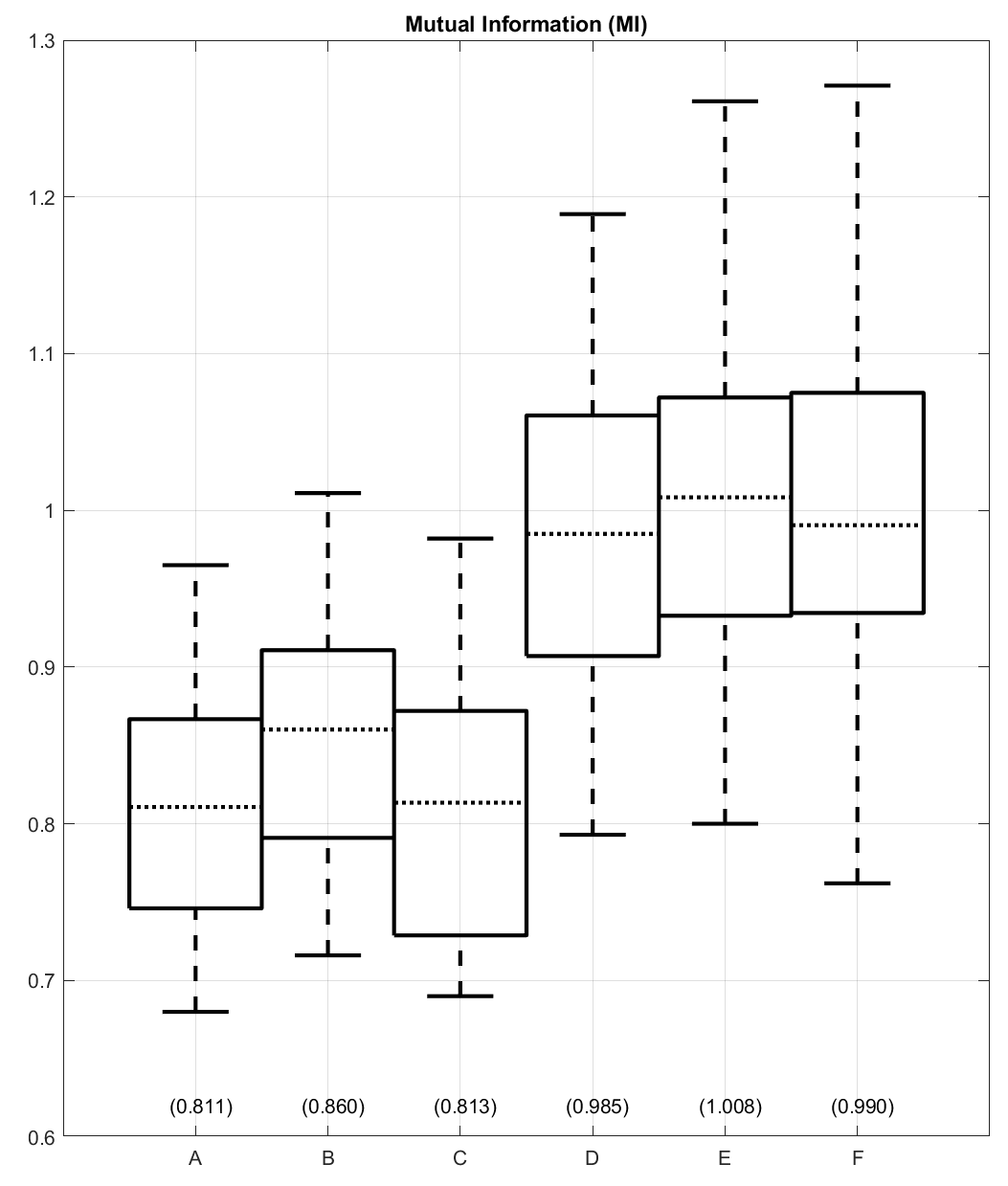


**Fig. S4.** Mutual Information (MI) comparison of all six methods. Method E achieves the highest MI.

**
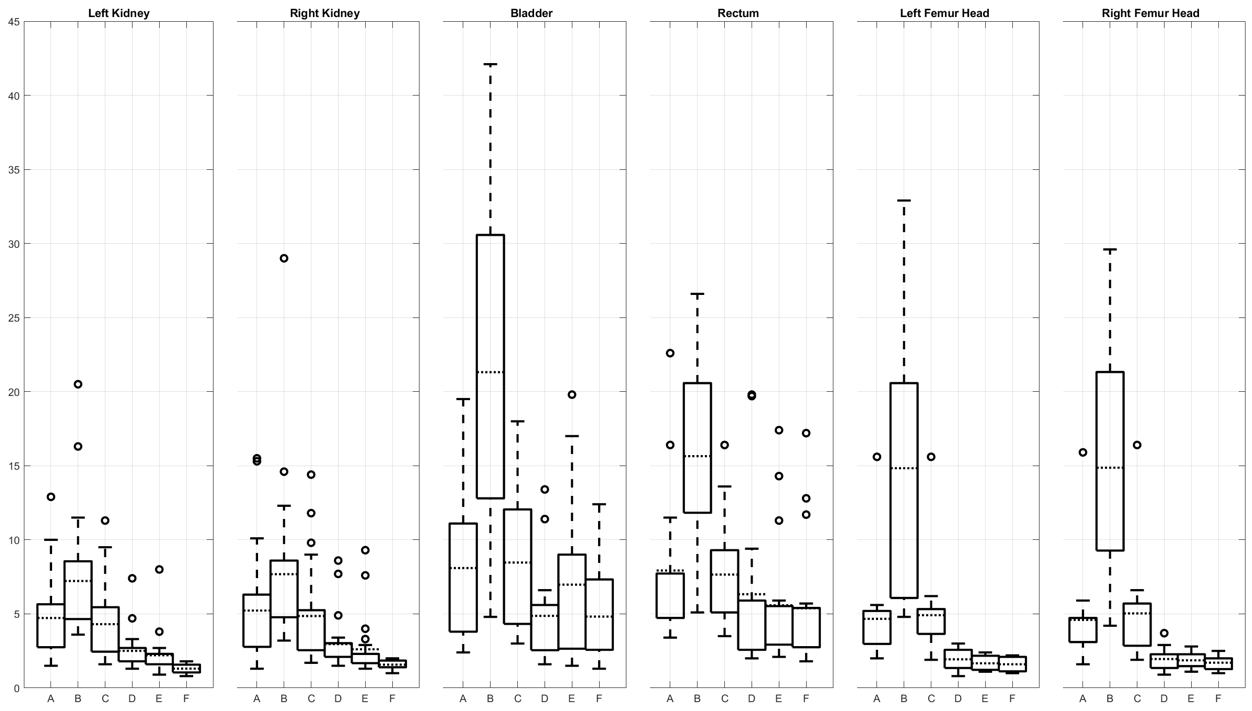
**

**Fig. S5.** MHD values comparison of ROI between the registered MR OP images from all six methods and the measured CT images. Note that methods D, E and F have smaller box height and lower mean values compared to methods A, B and C indicating better reproducibility and better registration accuracy.

**
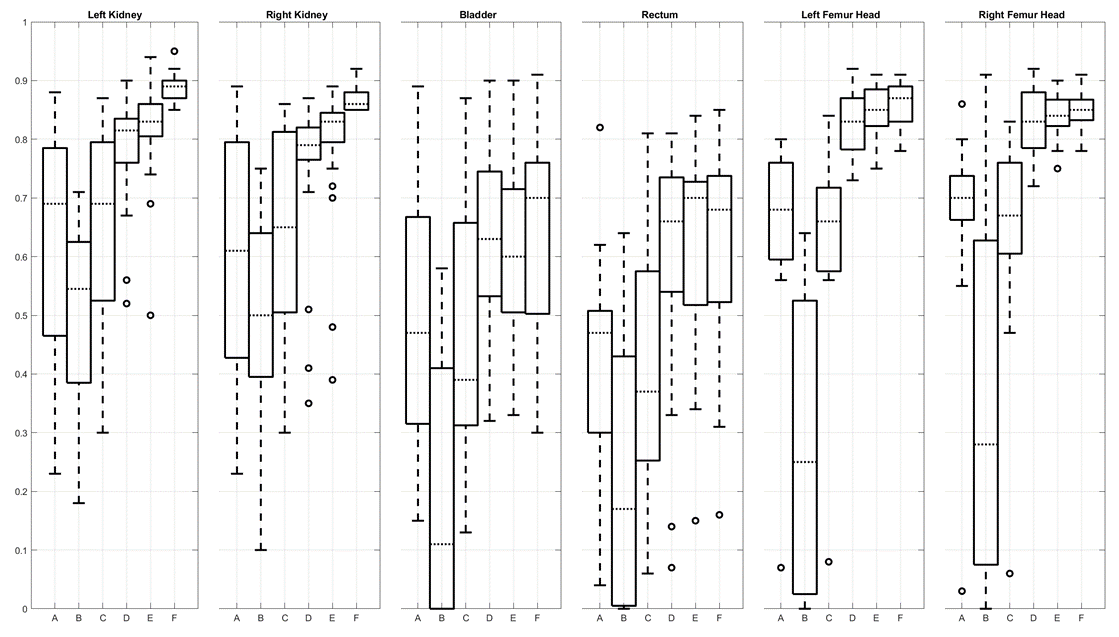
**

**Fig. S6.** DSI values comparison of ROI between the registered MR OP images from all six methods and the measured CT images. Note that methods D, E and F have smaller box height and higher mean values compared to methods A, B and C.

**
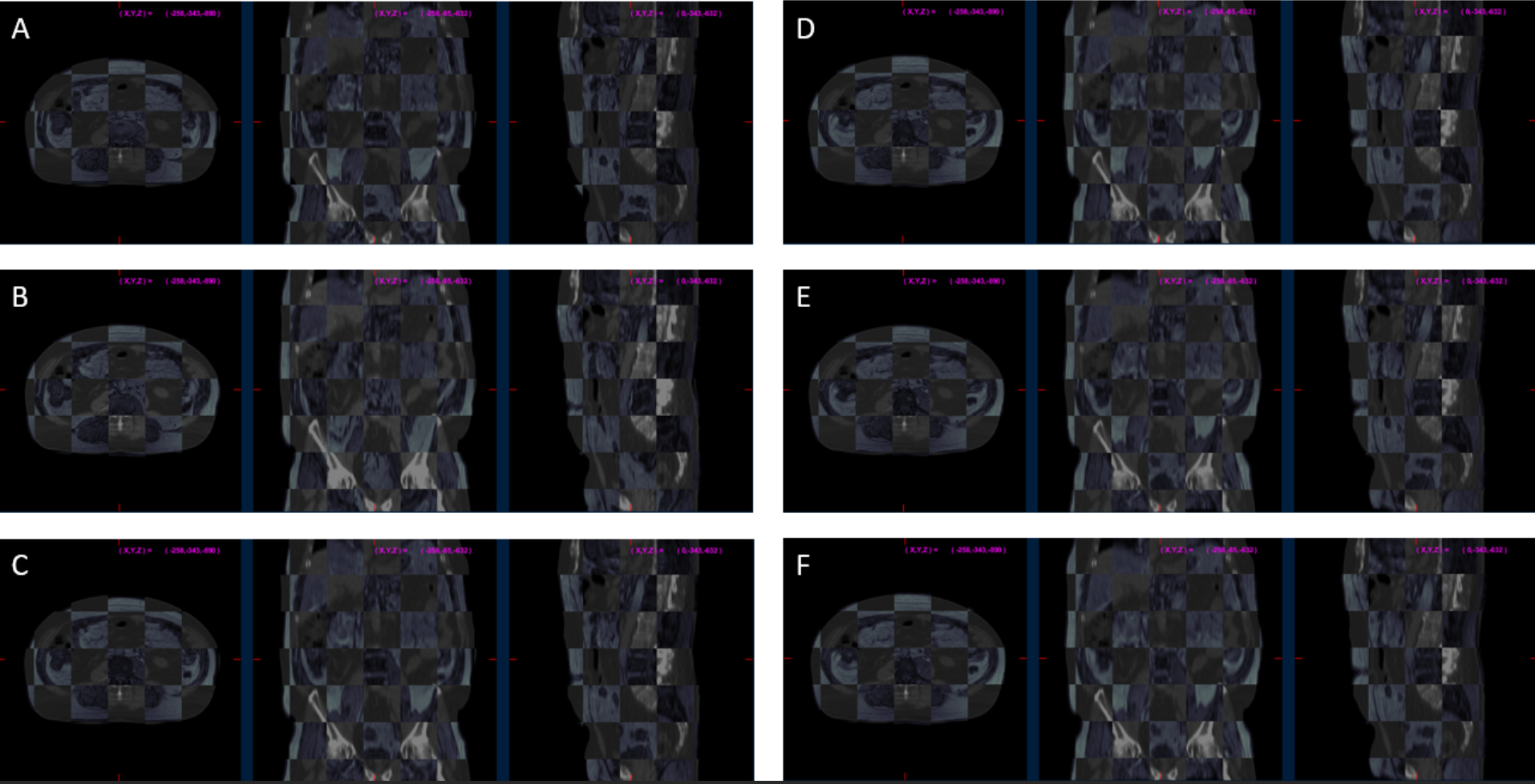
**

**Fig. S7.** Checkerboard composite images of CT and MR OP images from each method of a typical subject, with methods indicated in the upper left corner of each frame. The bone colormap – grayscale-like but with boosted blue intensity – is used for MR, and gray color map is used for CT to avoid big contrast differences that would impede visualization along the borders of the adjacent tiles.

**
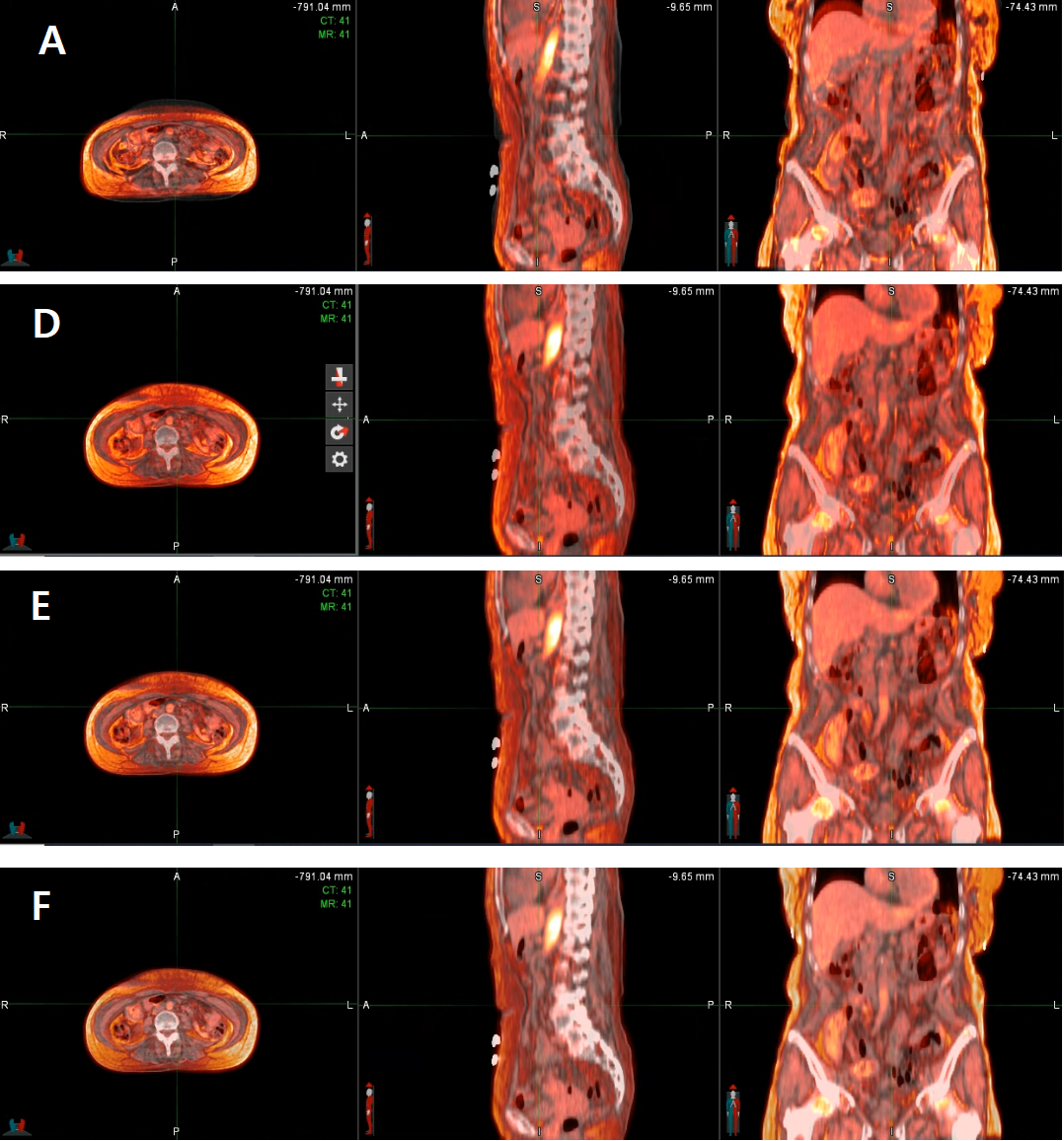
**

**Fig. S8.** Weighted fusion images of CT and baseline MR OP image (A) and the registered MR OP image using the proposed method (D), the MIM Maestro default method (E), and the MIM Maestro + SSD-based method (F) of a typical subject. MR images are shown using a hot-red color map and CT images are shown using a grayscale to keep the same setting for reviewer’s evaluation. Note that, it is extremely difficult to observe any significant difference, as these are comparisons between the sophisticated methods.
