# Supplementary material for: Abdominopelvic MR to CT registration using a synthetic CT intermediate
Source: J Appl Clin Med Phys. 2022 Aug 3;23(9):e13731. doi: 10.1002/acm2.13731 (PMC9512351; doi:10.1002/acm2.13731)
Supplement: Supplementary file 3 — Tables [file ACM2-23-e13731-s001.docx]

**Table S1**. P-values between the comparing methods for SSD value evaluation

| **Method**  **Mean±Std**  **(x 10^11^)** | **F**  1.24±0.31 | **D**  1.41±0.42 | **A**  1.75±0.49 | **C**  2.06±0.49 | **B**  3.96±0.12 | **E**  4.54±0.11 |
| --- | --- | --- | --- | --- | --- | --- |
| **p-value** | 0.025 | 0.0034 | 0.013 | 2.62 x 10^-10^ | 0.037 |  |

The left to right order of methods is organized by mean SSD, and the methods are F < D < A < C < B < E, from highest to lowest, or from best to worst registration accuracy. Values in the header row indicate the mean SSD value achieved after registering by the indicated method. P-values indicate the statistical significance of differences in SSD values between the successive methods. Note that only method D and F explicitly optimizes SSD. A lower SSD indicates a better registration so left-tailed tests are conducted. The t-test is constructed to test against the alternative hypothesis that the mean of the method in the present column is less (better) than the mean of the comparison method to the immediate right.

**Table S2**. P-values between the comparing methods for MAD value evaluation.

| **Method**  **Mean±Std** | **F**  64.8±5.7 | **D**  69.3±8.3 | **A**  77.0±8.7 | **C**  85.1±10.0 | **B**  124.1±13.8 | **E**  129.2±20.5 |
| --- | --- | --- | --- | --- | --- | --- |
| **p-value** | 0.0037 | 0.0016 | 0.0014 | 2.4 x 10^-10^ | 0.088 |  |

The left to right order of methods is organized by mean MAD, and the methods are F < D < A < C < B < E, from lowest to highest, or from best to worst registration accuracy. P-values indicate the statistical significance of differences in MAD values between the successive methods. The presentation is analogous to Table S1 noting that lower MAD values indicate better registration so left-tailed tests are conducted.

**Table S3.** P-values between the comparing methods for LPD value evaluation.

| **Method**  **Mean±Std** | **E**  0.605±0.089 | **D**  0.626±0.093 | **B**  0.637±0.093 | **F**  0.639±0.123 | **A**  0.642±0.094 | **C**  0.643±0.092 |
| --- | --- | --- | --- | --- | --- | --- |
| **p-value** | 0.13 | 0.28 | 0.45 | 0.46 | 0.47 |  |

The left to right order of methods is organized by mean LPD, and the methods are E < D < B < F < A < C, from lowest to highest, or from best to worst registration accuracy. P-values indicate the statistical significance of differences in LPD values between the successive methods. Note that only method B explicitly optimizes LPD. The presentation is analogous to Table S1 noting that lower LPD values indicate better registration so left-tailed tests are conducted.

**Table S4.** P-values between the comparing methods for MI value evaluation.

| **Method**  **Mean±Std** | **E**  1.008±0.109 | **F**  0.991±0.149 | **D**  0.985±0.113 | **B**  0.860±0.078 | **C**  0.813±0.090 | **A**  0.811±0.082 |
| --- | --- | --- | --- | --- | --- | --- |
| **p-value** | 0.21 | 0.43 | 5.74 x 10^-6^ | 0.0033 | 0.44 |  |

The left to right order of methods is organized by mean MI, and the methods are E > F > D > B > C > A, from highest to lowest, or from best to worst registration accuracy. P-values indicate the statistical significance of differences in MI values between the successive methods. Note that only method C explicitly optimizes MI. The presentation is analogous to Table S1 except that higher MI values indicate better registration so right-tailed tests are conducted.

**Table S5.** P-values between the comparing methods for MHD value (mm) evaluation for each ROI

| **Left Kidney** | | | | | | |
| --- | --- | --- | --- | --- | --- | --- |
| **Method**  **Mean±Std** | **F**  1.31±0.29 | **E**  2.21±1.36 | **D**  2.50±1.28 | **C**  4.31±2.44 | **A**  4.72±2.92 | **B**  7.22±4.14 |
| **p-value** | 5.01 x 10^-9^ | 0.15 | 2.46 x 10^-7^ | 0.21 | 0.00017 |  |

| **Right Kidney** | | | | | | |
| --- | --- | --- | --- | --- | --- | --- |
| **Method**  **Mean±Std** | **F**  1.57±0.29 | **E**  2.61±1.87 | **D**  2.96±1.72 | **C**  4.86±3.24 | **A**  5.22±3.72 | **B**  7.68±5.30 |
| **p-value** | 7.62 x 10^-10^ | 0.18 | 5.52 x 10^-6^ | 0.29 | 0.0015 |  |

| **Bladder** | | | | | | |
| --- | --- | --- | --- | --- | --- | --- |
| **Method**  **Mean±Std** | **F**  4.82±3.16 | **D**  4.87±3.39 | **E**  6.97±5.83 | **A**  8.09±4.84 | **C**  8.47±4.63 | **B**  21.3±12.2 |
| **p-value** | 0.48 | 0.015 | 0.24 | 0.39 | 1.90 x 10^-8^ |  |

| **Rectum** | | | | | | |
| --- | --- | --- | --- | --- | --- | --- |
| **Method**  **Mean±Std** | **F**  5.38±4.65 | **E**  5.58±4.77 | **D**  6.33±5.79 | **C**  7.65±3.61 | **A**  7.93±5.23 | **B**  15.65±6.26 |
| **p-value** | 0.44 | 0.28 | 0.20 | 0.39 | 0.000027 |  |

| **Left Femur Head** | | | | | | |
| --- | --- | --- | --- | --- | --- | --- |
| **Method**  **Mean±Std** | **F**  1.60±0.49 | **E**  1.67±0.49 | **D**  1.93±0.72 | **A**  4.67±3.24 | **C**  4.91±3.18 | **B**  14.83±9.42 |
| **p-value** | 0.30 | 0.027 | 3.61 x 10^-10^ | 0.39 | 4.23 x 10^-9^ |  |

| **Right Femur Head** | | | | | | |
| --- | --- | --- | --- | --- | --- | --- |
| **Method**  **Mean±Std** | **F**  1.71±0.45 | **E**  1.87±0.50 | **D**  1.95±0.73 | **A**  4.59±3.32 | **C**  5.03±3.46 | **B**  14.87±7.84 |
| **p-value** | 0.097 | 0.26 | 6.72 x 10^-10^ | 0.31 | 1.40 x 10^-8^ |  |

The left to right order of methods is organized by mean MHD, and, in general, the methods are F < E < D < A < C < B, from lowest to highest, or from best to worst registration accuracy. P-values indicating the statistical significance of differences in MHD values between the successive methods for each ROI. The presentation is analogous to Table S1 noting that lower MHD values indicate better registration so left-tailed tests are conducted.

**Table S6.** P-values between the comparing methods for DSI value evaluation for each ROI

| **Left Kidney** | | | | | | |
| --- | --- | --- | --- | --- | --- | --- |
| **Method**  **Mean±Std** | **F**  0.889±0.027 | **E**  0.814±0.083 | **D**  0.788±0.091 | **C**  0.640±0.182 | **A**  0.626±0.203 | **B**  0.491±0.163 |
| **p-value** | 1.59 x 10^-8^ | 0.068 | 2.50 x 10^-8^ | 0.36 | 0.0017 |  |

| **Right Kidney** | | | | | | |
| --- | --- | --- | --- | --- | --- | --- |
| **Method**  **Mean±Std** | **F**  0.871±0.022 | **E**  0.791±0.117 | **D**  0.755±0.132 | **C**  0.643±0.181 | **A**  0.612±0.206 | **B**  0.492±0.158 |
| **p-value** | 4.51 x 10^-10^ | 0.069 | 0.00015 | 0.20 | 0.0037 |  |

| **Bladder** | | | | | | |
| --- | --- | --- | --- | --- | --- | --- |
| **Method**  **Mean±Std** | **F**  0.648±0.182 | **D**  0.643±0.173 | **E**  0.625±0.167 | **A**  0.495±0.229 | **C**  0.463±0.228 | **B**  0.191±0.205 |
| **p-value** | 0.46 | 0.35 | 0.0048 | 0.30 | 0.0002 |  |

| **Rectum** | | | | | | |
| --- | --- | --- | --- | --- | --- | --- |
| **Method**  **Mean±Std** | **F**  0.624±0.189 | **E**  0.615±0.184 | **D**  0.579±0.226 | **A**  0.422±0.205 | **C**  0.407±0.512 | **B**  0.212±0.224 |
| **p-value** | 0.43 | 0.23 | 0.0087 | 0.39 | 0.0017 |  |

| **Left Femur Head** | | | | | | |
| --- | --- | --- | --- | --- | --- | --- |
| **Method**  **Mean±Std** | **F**  0.857±0.042 | **E**  0.844±0.046 | **D**  0.825±0.062 | **A**  0.647±0.181 | **C**  0.636±0.174 | **B**  0.266±0.251 |
| **p-value** | 0.12 | 0.068 | 1.23 x 10^-8^ | 0.41 | 4.91 x 10^-7^ |  |

| **Right Femur Head** | | | | | | |
| --- | --- | --- | --- | --- | --- | --- |
| **Method**  **Mean±Std** | **F**  0.852±0.036 | **E**  0.838±0.044 | **D**  0.831±0.056 | **A**  0.659±0.191 | **C**  0.635±0.190 | **B**  0.369±0.319 |
| **p-value** | 0.078 | 0.28 | 5.33 x 10^-9^ | 0.32 | 4.32 x 10^-5^ |  |

The left to right order of methods is organized by mean DSI, and, in general, the methods are F > E > D > A > C > B, from highest to lowest, or from best to worst registration accuracy. P-values indicating the statistical significance of differences in DSI values between the successive methods for each ROI. The presentation is analogous to Table S1 except that higher DSI values indicate better registration so right-tailed tests are conducted for this table.

**Table S7.** P-values between the comparing methods for sum-of-rank Mann-Whitney U test of Likert score evaluation.

| **Reviewer 1 (Attending musculoskeletal radiologist)** | | | | |
| --- | --- | --- | --- | --- |
| **Method**  **(Mean±Std)**  **[Median]** | **F**  (4.28±1.20)  [5] | **E**  (3.76±1.36)  [4] | **D**  (3.68±1.26)  [4] | **A**  (2.00±0.80)  [2] |
| **p-value** | 0.042 | 0.37 | 8.73 x 10^-6^ |  |

| **Reviewer 2 (Attending Nuclear Medicine Physician)** | | | | |
| --- | --- | --- | --- | --- |
| **Method**  **(Mean±Std)**  **[Median]** | **D**  (3.92±1.09)  [4] | **E**  (3.84±1.28)  [4] | **F**  (3.76±1.14)  [4] | **A**  (2.28±1.08)  [2] |
| **p-value** | 0.52 | 0.32 | 0.00005 |  |

| **Reviewer 3 (Fellow Nuclear Medicine Physician)** | | | | |
| --- | --- | --- | --- | --- |
| **Method**  **(Mean±Std)**  **[Median]** | **F**  (3.76±1.03)  [4] | **D**  (3.28±1.00)  [3] | **E**  (3.28±1.00)  [3] | **A**  (1.76±0.91)  [2] |
| **p-value** | 0.019 | 0.57 | 6.65 x 10^-6^ |  |

| **Reviewer 4 (Attending Nuclear Medicine Physician)** | | | | |
| --- | --- | --- | --- | --- |
| **Method**  **(Mean±Std)**  **[Median]** | **F**  (4.40±0.94)  [5] | **E**  (4.32±0.88)  [4] | **D**  (4.28±0.60)  [4] | **A**  (3.32±1.01)  [3] |
| **p-value** | 0.27 | 0.24 | 0.00021 |  |

| **Reviewer evaluation composite** | | | | |
| --- | --- | --- | --- | --- |
| **Method**  **(Mean±Std)**  **[Median]** | **F**  (4.05±1.13)  [4] | **E**  (3.80±1.21)  [4] | **D**  (3.79±1.09)  [4] | **A**  (2.34±1.13)  [2] |
| **p-value** | 0.048 | 0.35 | 2.54 x 10^-15^ |  |

The left to right order of methods is organized by sum of ranks Likert score, and considering a composite of all reviewers’ Likert score, the methods are F > E > D > A, from best to worst relative registration accuracy. P-values between the reference method (header) and the comparison method (immediate right header of the reference method) is evaluated using right-tailed, Mann-Whitney U test, because higher sum of rank value is better for Likert score. The top row indicates the specialty of the particular physician. The mean value is provided only as an alternative presentation metric of the sum of ranks, which is difficult to present.
